# Selective Catalytic Reduction of NO_x_ over Perovskite-Based Catalysts Using C_x_H_y_(O_z_), H_2_ and CO as Reducing Agents—A Review of the Latest Developments

**DOI:** 10.3390/nano12071042

**Published:** 2022-03-22

**Authors:** Ioannis V. Yentekakis, Amvrosios G. Georgiadis, Catherine Drosou, Nikolaos D. Charisiou, Maria A. Goula

**Affiliations:** 1 Laboratory of Physical Chemistry & Chemical Processes, School of Chemical & Environmental Engineering, Technical University of Crete, 73100 Chania, Greece; edrosou@isc.tuc.gr; 2 Foundation for Research and Technology—Hellas/Institute of Geoenergy (FORTH/IG), Technical University of Crete, Building M1, University Campus, 73100 Chania, Greece; 3 Laboratory of Alternative Fuels and Environmental Catalysis (LAFEC), Department of Chemical Engineering, University of Western Macedonia, Koila, 50100 Kozani, Greece; amvrosiosgeorgiadis@hotmail.com (A.G.G.); ncharisiou@uowm.gr (N.D.C.)

**Keywords:** NO_x_, perovskites, CO-SCR, H_2_-SCR, hydrocarbon-SCR

## Abstract

Selective catalytic reduction (SCR) is probably the most widespread process for limiting NO_x_ emissions under lean conditions (O_2_ excess) and, in addition to the currently used NH_3_ or urea as a reducing agent, many other alternative reductants could be more promising, such as C_x_H_y_/C_x_H_y_O_z_, H_2_ and CO. Different catalysts have been used thus far for NO_x_ abatement from mobile (automotive) and stationary (fossil fuel combustion plants) sources, however, perovskites demand considerable attention, partly due to their versatility to combine and incorporate various chemical elements in their lattice that favor deNO_x_ catalysis. In this work, the C_x_H_y_/C_x_H_y_O_z_^−^, H_2_^−^, and CO-SCR of NO_x_ on perovskite-based catalysts is reviewed, with particular emphasis on the role of the reducing agent nature and perovskite composition. An effort has also been made to further discuss the correlation between the physicochemical properties of the perovskite-based catalysts and their deNO_x_ activity. Proposed kinetic models are presented as well, that delve deeper into deNO_x_ mechanisms over perovskite-based catalysts and potentially pave the way for further improving their deNO_x_ efficiency.

## 1. Introduction

***DeNO_x_—general remarks***: The number of automobiles worldwide is constantly increasing, making the emission of CO, NO_x_ (x = 1, 2), hydrocarbons (HCs), and particulate matter (PM) in the atmosphere a major environmental problem of ever-increasing impact [1,2,3,4]. Similarly, increased energy demand for industry, home heating, etc., produced by stationary facilities, and still mainly based on fossil fuels, exacerbates the problem of air pollution in relation to these contaminants. Thereby, the regulations for emissions from stationary and mobile sources have become stringent while numerous technologies have been evolved in order to curb atmospheric pollution [1,2]. In general, heterogeneous catalysis for tackling environmental issues has been widely adopted as a low-cost, highly efficient, and selective technology for mitigating undesirable air pollutants that accompany energy production processes [5]. The prevalent heterocatalytic control technology of NO_x_ emissions (in excess of O_2_ in the gas stream) is called selective catalytic reduction (SCR); it is an end-of-pipe, after-treatment, process that selectively reduces NO_x_ emissions by means of different reducing agents such as NH_3_, urea, CO, H_2_, or HC/C_x_H_y_O_z_, using an appropriate catalyst [1,6,7,8,9,10,11,12,13,14]. Although NH_3_ and urea is currently the preferred choice for the SCR of NO_x_ applications in stationary power and chemical plants [6], reducing agents such as H_2_, light hydrocarbons, and CO have recently attracted intense interest, among other reasons, due to the fact that these components usually coexist in the exhaust gases [4,5,7,8,9,10,11,12,13,14,15].

Dispersed on mixed oxides, typically γ-Al_2_O_3_-(CeO_2_, La_2_O_3_, ZrO_2_, BaO, etc.) supports, noble metals such as Rh, Pd, Ir, and Pt have been demonstrated as the most efficient and tolerant to steam-induced lattice distortion and sulfur poisoning catalysts for the control of CO, HCs and NO_x_ emissions [15,16,17,18,19,20,21,22,23,24,25] and successfully applied for years in three-way catalytic converters (TWCs) technology [2]. However, despite intensive research efforts, such noble metal catalyst formulations, although very efficient in controlling emissions of stoichiometric gasoline engines (TWC conditions), have not been yet as effective as required for the control of non-stoichiometric engines emissions in order to be applicable in the case of lean-burn gasoline and diesel engines or in stationary fossil fuel combustion processes [1,7,13]. Bearing in mind that the use of precious metals is also associated with high costs and relatively poor stability, i.e., a propensity to particle agglomeration in the case of hot spots that often occur under real driving conditions (although means and methodologies for stabilizing dispersed catalyst nanoparticles against sintering have recently been discovered [26,27,28,29,30]), significant efforts have been put to the development and use of alternatives such as perovskite derived catalysts, due to their unique physicochemical properties, low cost, and favorable heat stability [31,32,33,34,35,36,37].

**Figure 1:**
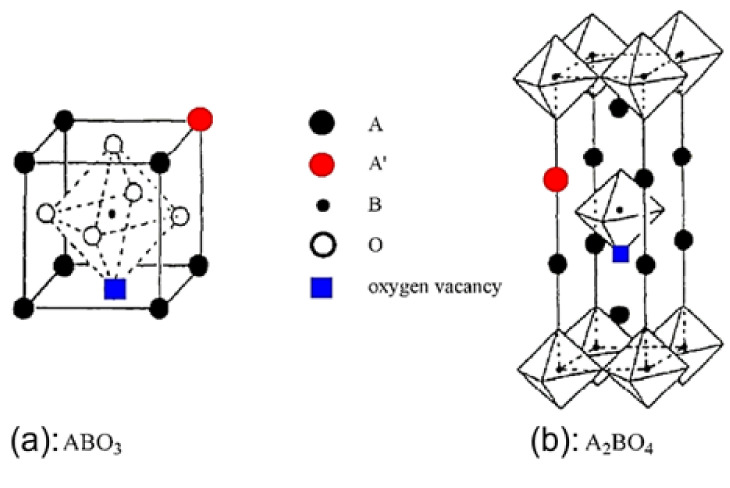
Ideal models of perovskite oxides with ABO_3_ and A_2_BO_4_ structure. The red dot represents the substitution of an A-site cation by a foreign one; the blue square represents the oxygen vacancies. The oxygen symbol is not shown in the A_2_BO_4_ structure for simplification. Reproduced with permission from Ref. [32]. Copyright 2014, ACS.

***Perovskite materials and their consideration in catalytic processes***: Perovskites is a class of oxides that has the structural formula ABO_3,_ and an ideal crystalline structure described as cubic from the Pm3m space group, as shown in Figure 1a. On the other hand, oxides with the structural formula A_2_BO_4_, which are composed of alternated ABO_3_ and AO layers (Figure 1b), have quite similar properties to ABO_3_ perovskites and are often called perovskite-like oxides [32]. Both oxide types are called hereinafter perovskites. In the structure of the perovskites, A is a large cation 12-fold coordinated with oxygen ions and located on the edge of the octahedron, while B is a smaller cation, six-fold coordinated with O^2−^ and located in the center of the octahedron (Figure 1a). The tolerance factor $t=\left(r_{A}+r_{O}\right)/\sqrt{2}\left(r_{B}+r_{O}\right)$ should lie within 0.75 < *t* < 1.0 in order to ensure perovskite matrix structure stability [32]. The A cation in the perovskite matrix can be an alkaline, alkaline earth, or lanthanide element, while the B cation can be an element from the 3d, 4d, or 5d configuration metals [32,33,34,35,36,37,38,39,40,41,42,43,44].

Perovskites are capable of partially substituting cations of A and/or B-sites by other cations with different or same valences (i.e., A_1−y_A′yB_1−x_B′_x_O_3±__δ_) to adjust their redox, bulk, and surface properties [38]. That said, with an appropriate combination of A′ and B′ metals the catalytic activity of a desired reaction can be readily tuned by modifying the perovskite chemical formula [33]. Indeed, besides their high thermal stability perovskites are characterized by some additional properties that make them favorable or even unique materials for several practical applications. For example, their high mixed electronic and ionic (O^2−^) conductivity makes them almost irreplaceable in electrocatalysis and solid oxide fuel cells (SOFCs) technology [34,45,46]; their intrinsic redox properties and oxygen ions mobility makes them beneficial materials in many heterocatalytic reaction systems, due to the sought after strong electronic metal–support interactions and oxygen ions back-spillover phenomena that accompany their use. The easily adjusted acid–base properties of perovskites are also key factors that make them favorable for regulating the activity and or selectivity of many catalytic reactions, including deNO_x_ [1,2,31,32,33,34,35,36,37,38,39,40,41,42]. It is also worth noting that the additional ability of partially substituting A and B-sites by other cations (i.e., A_1−y_A′yB_1−x_B′_x_O_3±__δ_), can provide a variety of (A, A′, B and B′ nature)-affected active sites for catalysis. For example, if La and a first series transition metal are selected for the A and B-site, respectively, high activity for NO reduction by CO can be achieved [43]. When lower valence ions are added in the A-site, which is typically occupied by a lanthanide, alkali, or alkaline earth [32,33,38,39,40,41,42,43,44], structural defections, as well as lattice distortion, can be seen, leading to an improvement in terms of catalytic behavior of B cation and lattice O_2_ mobility [47,48]. To illustrate this point doping with Ba^2+^, Sr^2+^, Rb^1+^, and Cs^1+^ can result in the formation of structural deficiencies (i.e., anionic vacancies) and an alteration in the oxidation state of the cations enhancing the perovskite’s catalytic performance.

An additional advantageous concept concerning the use of perovskite materials as metal catalyst nanoparticles supports is the so-called “redox exsolution”, discovered over the last decade [49], which opened new horizons and opportunities to heterogeneous catalysts design. Pioneers in the field, Nishihata et al. [50], used a Pd–perovskite catalyst to control automotive emissions and proved that the advanced electro-catalytic properties and high durability of this class of materials can be attributed to the utilization of metal nanoparticles exsolved from perovskite oxide lattices. The authors found that Pd can reversibly move into and out of the lattice of the perovskite while undergoing oxidation and reduction (as is usually the case in exhaust gas). This movement of Pd particles seemed to inhibit the growth of Pd nanoparticles and consequently led to improved catalytic activity for long-term use. However, despite the mounting interest in this method, a thorough understanding of how the perovskite supports and driving forces are combined is still lacking [51].

***The focus of the present review***: The aforementioned issues, and the fact that, to the best of our knowledge, there is no other literature report that focuses exclusively on the SCR of NO_x_ emphasizing these three types of reducing agents (i.e., C_x_H_y_/C_x_H_y_O_z_, H_2_, CO), we have gathered herein, exhaustively, recent relevant literature results on the subject, which are thoroughly and comparatively discussed in order to shed light on current developments and new perspectives.

## 2. Perovskite-Catalyzed SCR of NO_x_

Among the first to use perovskites in the SCR of NO_x_ was Buciuman et al. [47] who ascertained the superiority of the Sr-containing sample from a series of La_0.8_A_0.2_MnO_3_ perovskites (A = Cs, K, Ba, Sr) studied. He et al. [52] reported a strong dependence between the degree of x substitution and catalytic activity of a La_1−x_Sr_x_MO_3_ (M = Co_0.77_Bi_0.20_Pd_0.03_) perovskite under three-way catalysis (TWC) conditions, and Zhu et al. [53] showed a promising performance of La_2−x_Sr_x_CuO_4_ perovskites for the simultaneous removal of NO and CO under similar conditions. Fino et al. [54] proposed La_1.8_K_0.2_Cu_0.9_V_0.1_O_4_ as the best formulation for the simultaneous removal of NO_x_ and diesel particulate. In addition, considerable attention was paid to Cu-doped perovskites for the NO reduction by CO [43,55,56,57,58,59]. Noteworthy works have been carried out by Glisenti et al. [59] and Zhang et al. [43] who prepared B-site-Cu-doped perovskite catalysts to study the NO reduction by CO. With respect to A-site substitution, Ce, was thought to be the superior promoter as O_2_ desorption and reducibility seemed to increase after Ce was added into the perovskite structure, although excess amounts of Ce can result in the degradation of the perovskite structure compromising the catalytic performance of the material [60,61,62,63,64].

As we will see in the following sections, the reduction of NO using CO as a reducing agent on perovskite catalysts has been extensively studied under conditions of absence of O_2_ but very limited under conditions of excess O_2_ (i.e., SCR). However, the use of hydrogen as a reducing agent of NO_x_ under excess O_2_ conditions (H_2_-SCR) has been thoroughly studied providing encouraging results [65,66,67].

On the other hand, historically, the use of hydrocarbons as reducing agents for SCR of NO_x_ (C_x_H_y_-SCR) in O_2_-rich atmospheres has been investigated since the pioneering reports of Sato et al. [68]. In general, C_3_H_6_, C_3_H_8_, and CH_4_ are considered the most common reducing agents in the C_x_H_y_-SCR reaction in lean conditions [48,69,70]. Nevertheless, even though C_x_H_y_-SCR holds great promise, it is associated with poor activity in low-temperature domains. In this regard, O_2_-containing hydrocarbons (i.e., preferentially O_2_-rich C_x_H_y_O_z_) can be chosen as reducing agents to tackle this low-temperature inefficiency. Kucherov et al. [71] pioneered the use of ethanol as an effective reducing agent for NO reduction over Cu-ZSM-5 zeolites. The C_2_H_5_OH-SCR process was also investigated by Ukisu et al. [72] and Wu et al. [73] over Ag/Al_2_O_3_ catalysts. The latter group reported the superiority of enolic species over acetate species in generating —–CN/—–NCO species and resultantly promoting the activity. However, the narrow temperature window in terms of activity, which is related to these non-perovskite-type materials, is still observed compromising the catalytic activity of the catalysts. To this end, Wang et al. [74] synthesized perovskite-based catalysts to test their activity in the C_x_H_y_O_z_-SCR process under lean-burn conditions with methanol as the reducing agent.

A detailed analysis of the literature on the perovskites-catalyzed reduction of NO_x_ using hydrocarbons, hydrogen, or carbon monoxide as reducing agents follows. It is divided into three distinct chapters based on the means of reduction used. At the end of each chapter, a summary table is included that presents, in a comparative manner, the literature analyzed in each of the chapters.

### 2.1. Perovskite Catalysts in C_x_H_y_/C_x_H_y_O_z_-SCR of NO_x_

**Figure 2:**
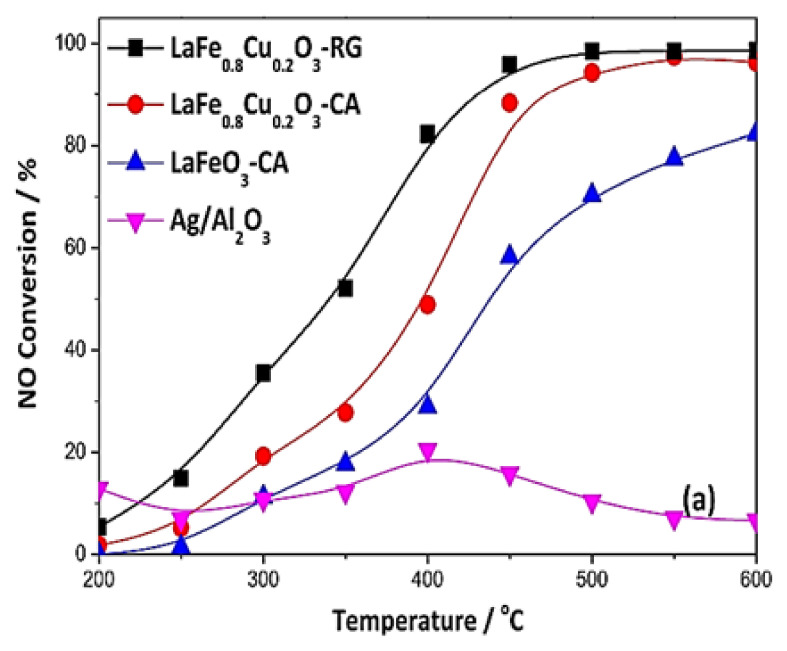
NO conversion performance versus temperature over different catalysts during the CH_3_OH-SCR of NO_x_. Reaction conditions: 1000 ppm NO, 3000 ppm CH_3_OH, and 8% O_2_. Reproduced with permission from Ref. [74]. Copyright 2019, Elsevier.

**Figure 3:**
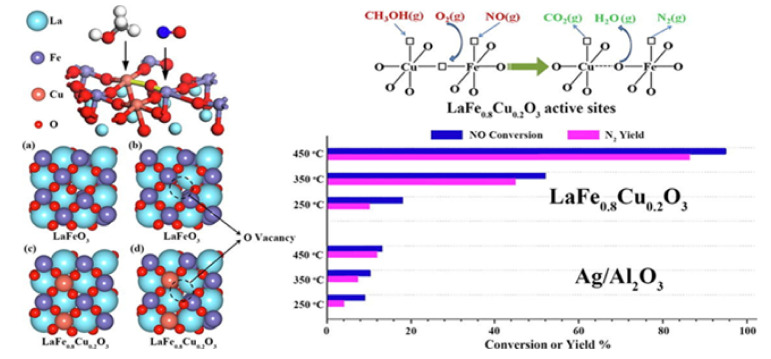
The deNO_x_ activity of LaFe_0.8_Cu_0.2_O_3_ perovskite in comparison to that of Ag/Al_2_O_3_ catalyst during CH_3_OH-SCR of NO_x_. The sites for methanol, O_2_, and NO adsorption/activation on the perovskite are also indicated. Reproduced with permission from Ref. [74]. Copyright 2019, Elsevier.

Wang et al. [74] investigated the SCR of NO by methanol (CH_3_OH) using a LaFe_0.8_Cu_0.2_O_3_ perovskite. The results were compared with those obtained on a Ag/Al_2_O_3_ reference catalyst, which is widely used in deNO_x_ applications. The said perovskite catalyst was prepared by a conventional citric acid (CA) complexation method, while the Ag/Al_2_O_3_ sample was prepared by wet impregnation. Furthermore, a high-surface-area nanoscale perovskite structure was produced by adapting the reactive grinding method (RG), which is a synthesis approach that is commonly used in metallurgy. The SCR activity tests were carried out in a tubular fixed bed quartz microreactor with a GHSV = 63,000 h^−1^. The feed mixture comprised of 1000 ppm NO, 3000 ppm CH_3_OH, 8% O_2,_ and He as balance gas. The catalyst physicochemical properties were explored by carrying out NO_ads_ + O_2ads_ TPD, XRD, H_2_-TPR, H_2_ physisorption, and isotopic exchange experiments. The LaFe_0.8_Cu_0.2_O_3_ sample modified using the RG method outperformed the other tested catalysts in terms of both NO conversion and N_2_ yield (Figure 2). Specifically, NO conversion of LaFe_0.8_Cu_0.2_O_3_/(RG) was almost 95% at 450 °C while N_2_ yield was approximately 93% as shown in Figure 2 and Figure 3; the latter figure also shows the perovskite sites on which the CH_3_OH, O_2_, and NO reactants are activated. The increased catalytic performance of LaFe_0.8_Cu_0.2_O_3_/RG was attributed to the higher surface area and subsequently to the increased number of surface-active sites available (Figure 3). Furthermore, a promoting effect regarding the formation of surface bounded O_2_ species was observed, probably due to the increased number of active redox sites resulting from the decrease in crystal domain size. The second-best catalyst was LaFe_0.8_Cu_0.2_O_3_/CA whereas the Cu-free LaFeO_3_/CA catalyst was third in activity order, offering maximum conversions that slightly exceeded 80% at the highest temperature (600 °C) investigated (Figure 2). The conventional Ag/Al_2_O_3_ catalyst performed poorly (close to inactive) for the entire temperature range under investigation (Figure 2).

The deNO_x_ performance of the catalysts was also investigated in the presence of CO_2_ and H_2_O in the reaction feed with the results showing an activity decrease of only ca. 10% in the presence of CO_2_ in the feed and a more profound decrease (ca. 20%) in the presence of H_2_O. Nevertheless, these inhibition effects were fully reversible when both CO_2_ and H_2_O were removed from the feed stream. Performing in situ DRIFTS studies the authors demonstrated the formation of methoxy species (−O−CH_3_) over Cu-containing samples. The presence of formohydroxamic acid and carboxylate was assigned to the reaction between the adNO_x_ nitrate/nitrite species detected on the surface of the Cu/Gr catalyst with the said methoxy species. With respect to the reference Ag/Al_2_O_3_ catalyst, a continuous accumulation of nitrate species over the alumina surface was noticed, upon which dehydration of methanol unequivocally ensued. This fact explained the absence of enolic intermediate of SCR over this conventional catalyst (Ag/Al_2_O_3_). The authors also proposed a reaction mechanism for LaFe_0.8_Cu_0.2_O_3_, supported by DFT calculations; the crucial step to producing N_2_ over this type of catalyst is C–N bond coupling along with the first H transfer (1.455 eV at the highest energy barrier) [74].

Teng et al. [75] conducted a joint experimental and theoretical study and proposed a system that combined enriching coal bed methane (CBM) with solar energy and SCR of NO_x_. The basic approach was that the enriched CBM could be used as a reducing agent in SCR with a La_0.8_Sr_0.2_MnO_3_ perovskite catalyst. Catalytic performance results showed that the CH_4_-SCR system exhibited the highest NO conversion (80%) with recorded outlet NO concentrations below 20 mg∙m^−3^. Regarding the numerical simulations, the Navier–Stokes equations were used with the hypothesis that density difference, caused by a temperature gradient, was the key parameter. The authors argued that the temperature gradient, caused by the exploitation of solar energy, can enrich CBM and subsequently more CH_4_ can be accumulated at the zone at increased temperature. In this regard, when the temperature difference was 150, the number of enriching units was estimated to be 200, which corresponded to a final CH_4_ mole fraction of 0.8.

The same group [76] used CH_4_ as a reductant to study the SCR of NO, though this time over an a-Al_2_O_3_-supported La_0.8_Sr_0.2_MnO_3_ perovskite-type catalyst. The precursor was prepared by a conventional sol–gel method, while the supported La_0.8_Sr_0.2_MnO_3_/a-Al_2_O_3_ catalyst was synthesized by a co-impregnation method. The perovskite structure of La_0.8_Sr_0.2_MnO_3_/a-Al_2_O_3_ was corroborated by XRD and SEM analysis. The inlet flue gas in the fixed bed reactor contained excess methane (CH_4_/NO = 1.2:1000 ppm NO, 1200 ppm CH_4_, 0–10% of O_2_ and N_2_ as carrier gas) to facilitate NO reduction, where the effect of temperature (600–900 °C) and resident time (τ = 1.0 s, 1.6 s, 2.2 s) was evaluated. It was shown that, in the absence of O_2_, methane can effectively convert NO (i.e., above 90%) over the La_0.8_Sr_0.2_MnO_3_/a-Al_2_O_3_. On the other hand, in the presence of O_2_, the NO conversion was positively correlated with resident time. A positive correlation was also observed between NO conversion and temperature when the O_2_ content ranged from 0% to 3%. Furthermore, at high reaction temperatures, moderate O_2_ concentration was found to promote the NO reduction by filling the O_2_ vacancies in the lattice. Interestingly, the supported catalyst had a decent performance for the broadest range of O_2_ concentrations, while it outreached 90% of NO conversion when the O_2_ concentration ranged from 4% to 6%. The authors concluded that the optimal experimental condition was: 2.2 s of residence time, 4–6% of O_2_ concentration in feed gas, and 800 °C of reaction temperature.

**Figure 4:**
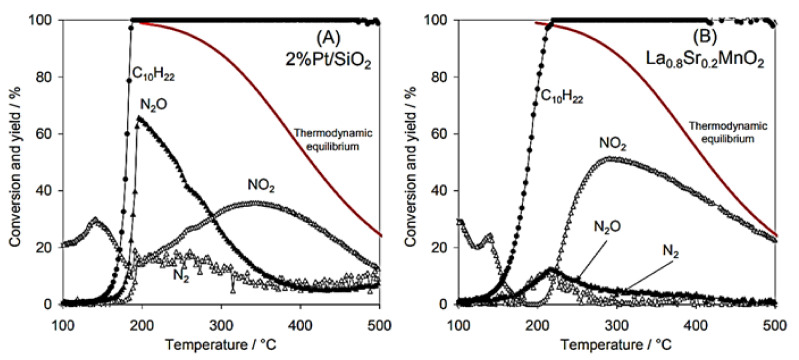
Reactant (C_10_H_22_) conversion and product (N_2_, N_2_O, NO_2_) yields for Pt/SiO_2_ (**A**) and La_0.8_Sr_0.2_MnO_3_ (**B**) catalysts (feed gas: 400 ppm NO, 240 ppm C_10_H_22_, 1.5 vol% H_2_O, and 9 vol% O_2_). Reproduced with permission from Ref. [77]. Copyright 2014, Elsevier.

Giroir-Fendler et al. [77] evaluated the catalytic activity of LaMnO_3_ and partially substituted La_0.8_A_0.2_MnO_3_ (A = K, Sr) perovskites for the SCR of NO using decane (C_10_H_22_) as reductant, as well as NO oxidation and C_10_H_22_ oxidation. A 2 wt% Pt/SiO_2_ sample was also used as a reference catalyst for comparison. The perovskite materials were synthesized by a complexation route. The gas mixture comprised of 400 ppm(v) NO, 240 ppm(v) C_10_H_22_, 1.5 vol% H_2_O, and 9 vol% O_2_ (WGHSV = 36,000 mL∙g^−1^∙h^−1^) resembling a diesel exhaust gas, and the light-off behavior of the catalytic systems was acquired in the temperature range 100 to 500 °C (Figure 4). With respect to the ref. [2] wt% Pt/SiO_2_ catalyst, 50% conversion was achieved at 180 °C which was 50 °C higher compared to the corresponding temperature shown during the C_10_H_22_ oxidation experiments (Figure 4A). The presence of NO did not affect the oxidation of C_10_H_22_ when the La_0.8_Sr_0.2_MnO_3_ catalyst was used (Figure 4B). Catalytic tests in the absence of NO at 190 °C provided a similar T_50_ value. Interestingly, C_10_H_22_ conversion for both Pt/SiO_2_ and La_0.8_Sr_0.2_MnO_3_ catalysts reached 100% at 200 °C (Figure 4).

**Figure 5:**
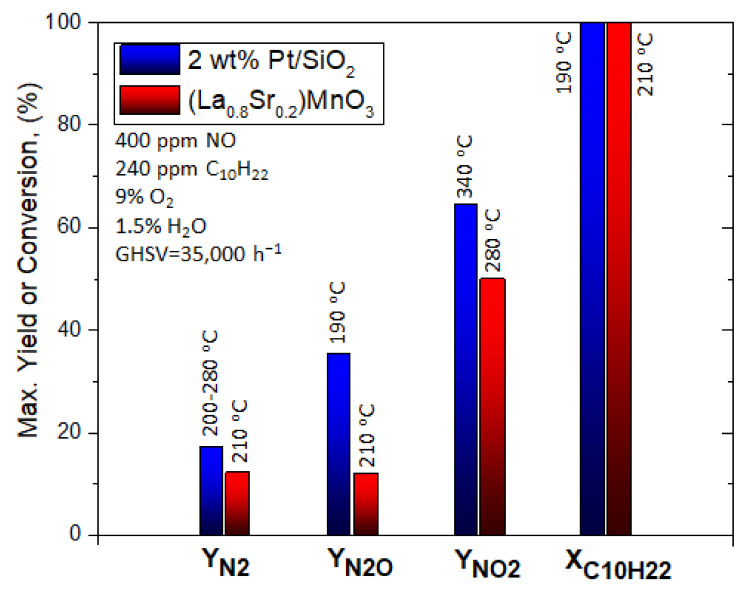
Comparison of the maximum N_2_, N_2_O, NO_2_ yields, and NO conversions and the corresponding temperatures these maxima appeared at for the C_10_H_22_-SCR of NO_x_ over a noble metal-based catalyst and a noble metal-free perovskite catalyst. Reproduced with permission from ref. [77]. Copyright 2014, Elsevier.

A closer look at the behavior of Pt/SiO_2_ catalyst under SCR conditions (Figure 4A) shows that both reduction of NO to N_2_O and C_10_H_22_ oxidation initiated at the same temperature, while the maximum N_2_O yield (i.e., 65%) was obtained at 195 °C when the conversion of C_10_H_22_ was 100%. The amount of N_2_ produced was lower compared to that of N_2_O, both reaching maximum yields of 18% and 65%, respectively, at ~200 °C. Exceeding 200 °C, N_2_ and N_2_O yields dropped, though, NO conversion to NO_2_ showed an increasing trend and reached a maximum of 37% at 370 °C. The maximum NO_2_ yield was lower and shifted to higher temperatures when NO oxidation took place in the absence of C_10_H_22_. In addition, the NO_2_ yield was below the thermodynamic equilibrium curve even at the highest temperature of 500 °C reached (Figure 4A). The La_0.8_Sr_0.2_MnO_3_ catalyst showed a relatively different catalytic behavior than Pt/SiO_2_. A less competitive relationship between NO and C_10_H_22_ was observed for temperatures lower than 200 °C, and thereby NO conversion was not favored toward C_10_H_22_ oxidation. The maximum values of N_2_ and N_2_O yields (almost 13%) at 210 °C were obtained when the conversion of C_10_H_22_ was 100%. NO_2_ production started at 200 °C and reached a maximum value of 50% at 290 °C. Regarding the maximum yield of NO_2_, it was lower compared to that of the NO oxidation experiment in the absence of C_10_H_22_, however, it was recorded at almost the same temperature ca. 285–290 °C. The fact that the NO/C_10_H_22_ system exhibited lower maximum NO_2_ yield was ascribed to the parallel oxidation reactions of C_10_H_22_ and NO over different redox systems (Mn^3+^/Mn^2+^ and/or Mn^4+^/Mn^3+^). The said redox systems were considered as the active species participating in the Mars–van Krevelen mechanism, which is a widely accepted mechanism for hydrocarbons oxidation using mixed oxide catalysts [78,79]. The perovskite (La_0.8_Sr_0.2_MnO_3_ catalyst) showed better NO → NO_2_ oxidation activity and thus allowed a closer approximation of the thermodynamic equilibrium of this reaction compared to the Pt-based catalyst (Figure 4). Finally, the authors concluded that the La_0.8_Sr_0.2_MnO_3_ perovskite could be a promising alternative, noble metal-free catalyst for NO_x_ emission control (Figure 5). Table 1 synopsizes the main literature studies analyzed in Section 2.1.

### 2.2. Perovskite Catalysts in H_2_-SCR of NO_x_

Efstathiou and co-workers [9] using a ceramic method prepared a La_0.7_Sr_0.2_Ce_0.1_FeO_3_ solid in which the major crystal phases detected by XRD were LaFeO_3_ and SrFeO_3−x_ perovskite structures and the oxidic phases CeO_2_ and Fe_2_O_3_ (i.e., not a pure perovskite but a mixed oxide material). The material was used as support for the preparation of a 1 wt% Pt content catalyst, by wet impregnation (1 wt% Pt/La_0.7_Sr_0.2_Ce_0.1_FeO_3_), while counterpart catalysts, namely 1 wt% Pt content Pt/CeO_2_, Pt/Fe_2_O_3,_ and Pt/SiO_2_, were also prepared for the sake of comparison. The H_2_-SCR deNO_x_ performance of the Pt/La_0.7_Sr_0.2_Ce_0.1_FeO_3_ catalyst, using a 0.25% NO/1% H_2_/5% O_2_/balance He at a WGHSV of 40,000 mL∙g^−1^∙h^−1^ (GHSV = 80,000 h^−1^), found to transcend that of the other tested catalysts: a maximum NO conversion of 83% with a N_2_-selectivity as high as 93% was achieved at 150 °C; corresponding maximum values for Pt/SiO_2_, Pt/CeO_2_, and Pt/Fe_2_O_3_ catalysts were X_NO_ = 82%/S_N2_ = 65% (at 120 °C), X_NO_ = 82%/S_N2_ = 43% (at 150 °C), and X_NO_ = 16%/S_N2_ = 5% (at 200 °C), respectively. Notably, for the optimal Pt/La_0.7_Sr_0.2_Ce_0.1_FeO_3_ catalyst, the addition of 5% H_2_O in the feed stream at 140 °C resulted in some widening of the operating temperature window with considerable NO conversion and N_2_-selectivity values, while no degradation effects were observed on its stability for 20 h time-on-stream.

**Figure 6:**
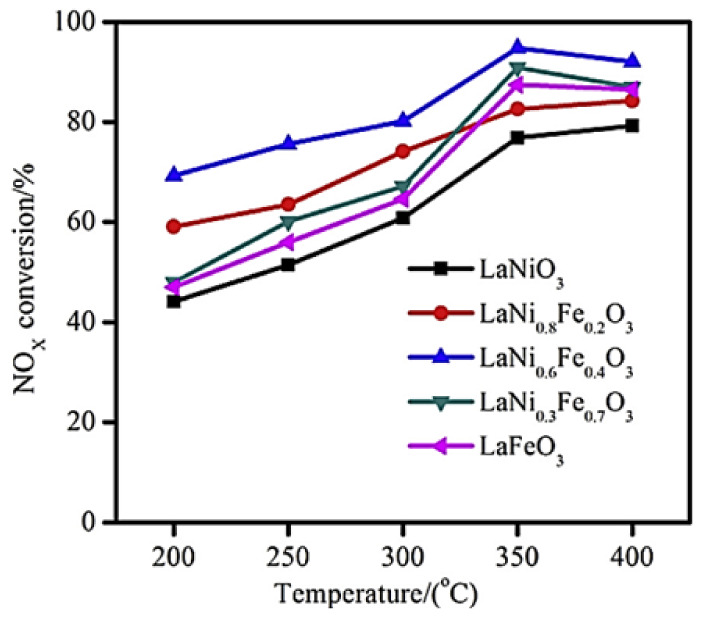
Effect of temperature on NO_x_ conversion efficiency of LaNi_1−x_Fe_x_O_3_ perovskite catalysts during H_2_-SCR of NO_x_. Reproduced with permission from Ref. [80]. Copyright 2014, Elsevier.

Luo et al. [80] prepared, via a sol–gel method, a series of LaNi_1−x_Fe_x_O_3_ (x = 0.0, 0.2, 0.4, 0.7, 1.0) perovskites to study the SCR of NO_x_ by H_2_ at temperatures between 200 and 400 °C in a fixed bed reactor (500 mg catalyst) using a 500 ppm NO, 3.5 vol% H_2_, 8 vol% O_2_ (balance N_2_) gas feed composition with a total flow rate of 600 mL min^−1^. Sulfur-aging and regeneration treatments were also involved in their study. They found that Fe-doping of the base LaNiO_3_ perovskite results in a better NO_x_ removal (Figure 6), and high structural stability.

**Figure 7:**
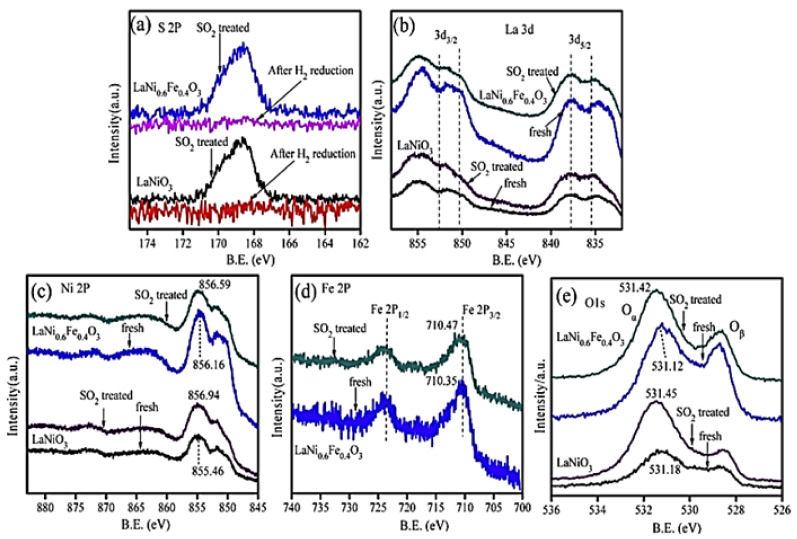
XPS analysis of LaNi_1−x_Fe_x_O_3_ in the (**a**) S 2p, (**b**) La 3d, (**c**) Ni 2p, (**d**) Fe 2p, and (**e**) O 1s. Reproduced with permission from Ref. [80]. Copyright 2014, Elsevier.

H_2_-TPR analysis showed that partial substitution of Ni by Fe results in better reducibility of nickel particles (Ni^3+^ → Ni^2+^), which in turn play a critical role in promoting NO-SCR. The perovskite LaNi_0.6_Fe_0.4_O_3_ with the best reducibility characteristics was the best overall in deNO_x_ performance (Figure 6). Regarding perovskite stability, N_2_ physisorption and SEM analysis showed that the partial substitution of nickel with appropriate amounts of Fe can lead to enhanced surface area as well as thermal stability. Finally, the bulk LaNiO_3_ and LaNi_0.6_Fe_0.4_O_3_ were tested toward their sulfur resistance and regeneration ability. Results showed that the presence of SO_2_ resulted in lower NO_x_ conversion in both cases. However, both samples were capable of being regenerated after 12-h long H_2_ treatment. XPS results (Figure 7) suggested the predominance of sulfate species formed on the active nickel components, whereas the addition of Fe significantly affected the sulfation process, leading to enhanced sulfur resistance.

**Figure 8:**
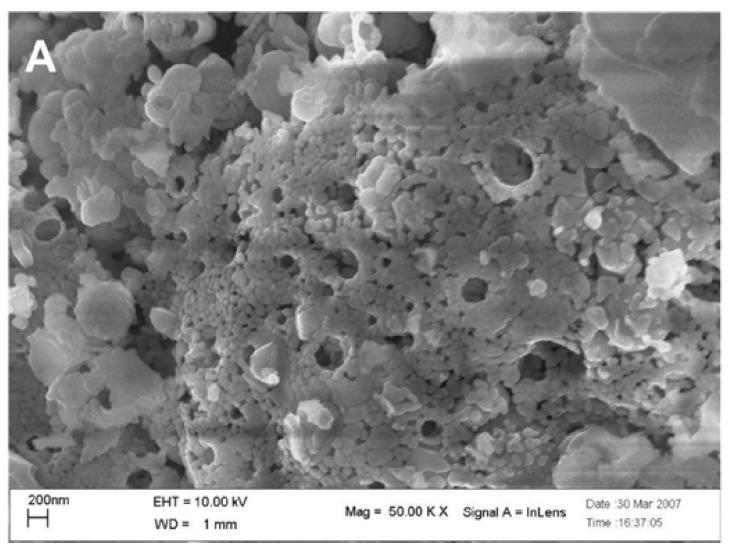
Field emission SEM micrograph of the La_0.8_Sr_0.2_FeO_3_ catalyst crystals. Reproduced with permission from Ref. [81]. Copyright 2010, Elsevier.

**Figure 9:**
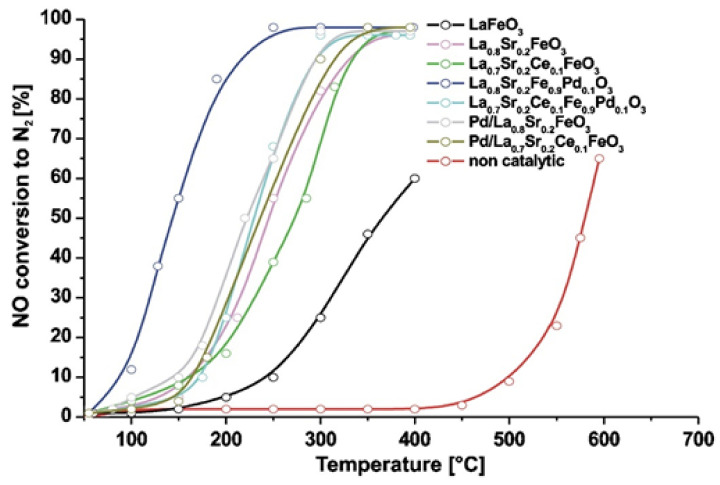
Comparison of the NO conversion light-off performance of all synthesized perovskite-type catalysts under 1000 ppmv NO, 4000 ppmv H_2_, 0% O_2_, balance He, WGHSV = 180,000 mL∙g^−1^∙h^−1^ feed conditions. Reproduced with permission from Ref. [81]. Copyright 2010, Elsevier.

Using a cost-effective solution combustion synthesis (SCS) method, Furfori et al. [81] prepared a series of perovskites and Pd-promoted (via wet impregnation) perovskite-type catalysts belonging to the LaFeO_3_ group in order to evaluate their catalytic performance and decipher H_2_-SCR of NO_x_ mechanisms in the absence and presence of O_2_. The synthesized catalysts were La_0.8_Sr_0.2_FeO_3_, Pd/La_0.8_Sr_0.2_FeO_3_, La_0.8_Sr_0.2_Fe_0.9_Pd_0.1_O_3_, La_0.7_Sr_0.2_Ce_0.1_FeO_3_, Pd/La_0.7_Sr_0.2_Ce_0.1_FeO_3_, La_0.7_Sr_0.2_Ce_0.1_Fe_0.9_Pd_0.1_O_3_, and bulk LaFeO_3_; Figure 8 shows a field emission scanning electron micrograph of the La_0.8_Sr_0.2_FeO_3_ sample appearing as a very foamy structure, a structure that was found to be representative of all perovskites prepared by the SCS method with a specific surface area, ~10 m^2^_∙_ g^−1^ on average. The catalytic tests performed in a fixed bed reactor at the temperature range of 25–400 °C, using a gas feed consisted of 1000 ppm NO, 4000 or 10,000 ppm H_2_, 0 or 5% O_2_, balance He, at WGHSVs = 180,000, 270,000 and 360,000 mL∙g^−1^∙h^−^^1^ (GHSV = 20,000, 30,000, and 40,000 h^−1^). In the absence of O_2_ in the feed stream, the most promising catalyst that outperformed all other catalysts was found to be La_0.8_Sr_0.2_Fe_0.9_Pd_0.1_O_3_ (Figure 9), which was then further studied in the presence of 5% O_2_ at three different WGHSVs, and at a higher H_2_ concentration in the feed (10,000 ppm). Up to 75% NO conversion toward N_2_ at a temperature as low as 125 °C for WGHSV = 180,000 mL∙g^−1^∙h^−^^1^ was achieved. For the highest space velocity value, the maximum NO conversion to N_2_ was reduced to ~55% while the corresponding temperature was shifted to a value ~10 °C higher. As the temperature increased, the selectivity to N_2_ gradually decreased due to the favorable formation of N_2_O and NO_2_, a behavior typical of SCR processes. Regarding the deNO_x_ reaction mechanism, H_2_-TPR and other characterization results allowed the authors to conclude that both the availability of oxygen vacancies (suitable for NO adsorption) and the reducibility of the B-sites play a critical role in the catalytic activity of the perovskites. Finally, the authors stated that the results, although promising, do not yet meet industry demands.

**Figure 10:**
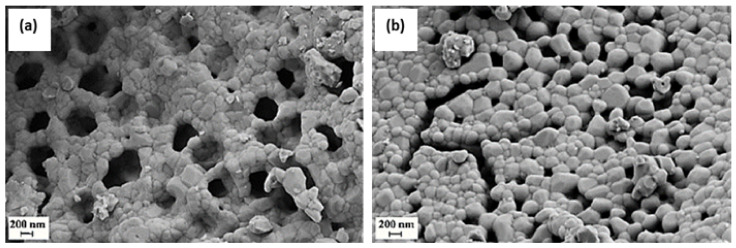
SEM images of the perovskite surface LFC_(0.3)_-Pd (**a**) and LFC_(0.475)_-Pd (**b**) after calcination in air at 900 °C/3 h. Reproduced with permission from Ref. [67]. Copyright 2010, Elsevier.

Mondragon Rodriguez and Saruhan [67] studied the effect of Fe/Co-ratio on the phase composition of LaFe_0.95−x_Co_x_Pd_0.05_O_3_ (x = 0.475, 0.4, 0.3) perovskite catalysts and then evaluated their behavior with respect to H_2_-SCR of NO_x_. The catalysts under consideration were prepared by the so-called citrate method. Three different feed stream composition protocols were adopted during catalyst evaluation tests in a tubular fixed bed reactor operated at 1 bar. In the first one the feed gas comprised of 0.072% NO, 5% O_2_, 1% H_2_, He balance. The second experimental protocol included the addition of 7.2% H_2_O and 7.2% CO_2_ to the mixture (0.072% NO, 5% O_2_, 1% H_2_, 7.2% H_2_O), while the third one included the addition of 0.25% CO instead of H_2_O, and CO_2_ (0.072% NO, 5% O_2_, 1% H_2_, 0.25% CO). The WGHSV in all experiments was kept at 55,000 mL∙g^−1^∙h^−^^1^ and the reaction temperature ranged from 50 to 400 °C. All tested samples were characterized using the TPR, EDS, FESEM, XPS, XRD, and DSC techniques. Results from the first experimental protocol suggested that LaFe_0.475_Co_0.475_Pd_0.05_O_3_ and LaFe_0.65_Co_0.3_Pd_0.05_O_3_ exhibited the best (i.e., 79% NO conversion at 180–230 °C) and second-best (i.e., 74% NO conversion at 200 °C) catalytic performance, respectively. Representative field emission SEM images of these two materials are depicted in Figure 10.

**Figure 11:**
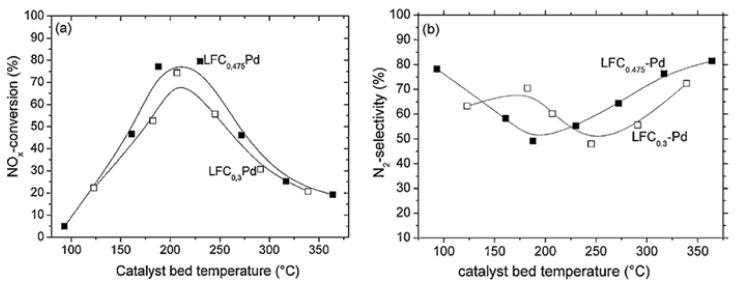
NO_x_ conversion (**a**) and N_2_-selectivity (**b**) of LaFe_0.475_Co_0.475_Pd_0.05_O_3_ and LaFe_0.65_Co_0.3_Pd_0.05_O_3_ perovskite catalysts. The samples were calcined at 900 °C in air for 3 h. Reproduced with permission from Ref. [67]. Copyright 2010, Elsevier.

Figure 11a shows that the NO_x_ conversion of LaFe_0.475_Co_0.475_Pd_0.05_O_3_ and LaFe_0.65_Co_0.3_Pd_0.05_O_3_ followed a typical volcano-type behavior [67], indicating the existence of competing redox reactions which might entail the dissociation of NO into adsorbed N and O species, as also proposed by Burch and Coleman [82] upon investigating the H_2_-SCR of NO_x_ over platinum group metal catalysts dispersed on traditional oxide supports. The optimum operating temperature was between 200 °C and 250 °C (Figure 11a). By decreasing the Co-content of the La-containing perovskites, the selectivity of N_2_ shifted to higher temperatures (Figure 11b). Nevertheless, LaFe_0.475_Co_0.475_Pd_0.05_O_3_ was found to produce slightly more N_2_O (35 ppm) between 160 °C and 240 °C, in comparison to LaFe_0.65_Co_0.3_Pd_0.05_O_3_. Interestingly the latter catalyst reduced less NO_x_ compared to LaFe_0.475_Co_0.475_Pd_0.05_O_3_ suggesting that either Co or Co-species can promote the development of active sites, which participate in the NO_x_ reduction. Additionally, with respect to the bimetallic particles (i.e., Co and Pd), they may be involved in NO-dissociation and chemisorption, N_2_O formation, and H_2_-chemisorption. The authors also examined the effect of CO_2_ and H_2_O given that they are always involved in the exhausts of any combustion engine and can affect the catalyst performance during the H_2_-SCR of NO_x_ reaction. Results from the second series of these catalytic tests showed that the NO conversion dropped when the Co amount in the structure of the perovskite decreased, whereas N_2_ selectivity remained constant. When the temperature was lower than 250 °C the NO_x_ reduction performance decreased, while above 250 °C the NO_x_ conversion was maintained. Competitive adsorption of H_2_O and NO molecules on the active sites of the perovskite was observed, affecting the NO_x_ conversion, and increasing the formation of N_2_O. In addition, as the temperature increased above 250 °C lower amounts of N_2_O were produced over the LaFe_0.65_Co_0.3_Pd_0.05_O_3_ sample [67]. The results from the experiments involving CO_2_ and H_2_O in the feed gas indicated that H_2_O molecules can easily dissociate at increased temperatures on the catalyst surface, facilitating the formation of adsorbed H species, which may eventually lead to higher concentrations of N adsorbed species via the reaction NO_ads_ + H_ads_ → N_ads_ + OH_ads_ as also proposed by Dhainaut et al. [83] upon studding the H_2_-SCR of NO_x_ on Pd/LaCoO_3_ catalysts. Resultantly, more molecular N_2_ was formed due to the reaction between two neighboring chemisorbed N atoms on the surface of the LaFe_0.65_Co_0.3_Pd_0.05_O_3_. The reason why these reactions occurred on catalyst Pd, Pd-Fe, or/and Pd-Co-surface was that the Pd-free sample exhibited no activity for the H_2_-SCR of NO_x_ reaction. Agreeing with Dhainaut et al. [83], the authors stated that the physicochemical properties of Pd were strongly affected by its interaction with La [67]. On the other hand, the LaFe_0.475_Co_0.475_Pd_0.05_O_3_ catalyst displayed improved NO_x_ conversion in the presence of H_2_O vapor in the feed. Results suggested that 20% more NO_x_ was reduced for the H_2_O-containing mixture in comparison to that or H_2_O-free mixture (dry conditions) at 350 °C [67]. Decreased amount of N_2_O was also observed with H_2_O vapor in the reaction that took place below 250 °C, while at higher temperatures N_2_O formation was almost constant. Under these conditions, H_2_O was more likely to be dissociated on the LaFe_0.475_Co_0.475_Pd_0.05_O_3_ surface promoting the formation of molecular N_2_ and lowering the chance of N_2_O formation. The authors suggested that the improved N_2_ selectivity of Pd-La perovskites was probably assigned to the existence of alloy compounds (e.g., Pd_3_La) as was also reported by others [66,84]. The effect of CO in the feed was also examined in the third experimental protocol and it was found that both the N_2_ selectivity and the NO_x_ conversion for LaFe_0.475_Co_0.475_Pd_0.05_O_3_ and LaFe_0.65_Co_0.3_Pd_0.05_O_3_ perovskites were negatively affected by CO presence [67]. LaFe_0.475_Co_0.475_Pd_0.05_O_3_ exhibited higher N_2_ selectivity compared to that of LaFe_0.65_Co_0.3_Pd_0.05_O_3_ reaching 54% at 180 °C and 46% as the temperature ranged from 210 to 290 °C. Increasing the Fe-content led to decreased NO_x_ reduction performance for temperatures above 225 °C. Regarding the LaFe_0.65_Co_0.3_Pd_0.05_O_3_ catalyst, lower N_2_-formation rates were observed while the maximum value of N_2_ selectivity was 34% at 237 °C. A 10% decrease was noticed at higher temperatures (i.e., 340 °C) because of the relatively high N_2_O concentration formed. Results suggested that higher Co-content in the catalyst, resulted in the formation of perovskite phase, as corroborated by in situ XRD measurements. Another conclusion made was that the new intermetallic phases, which can be formed between the metallic Fe, Pd, and Co perovskite components, were more resistant to CO poisoning.

**Figure 12:**
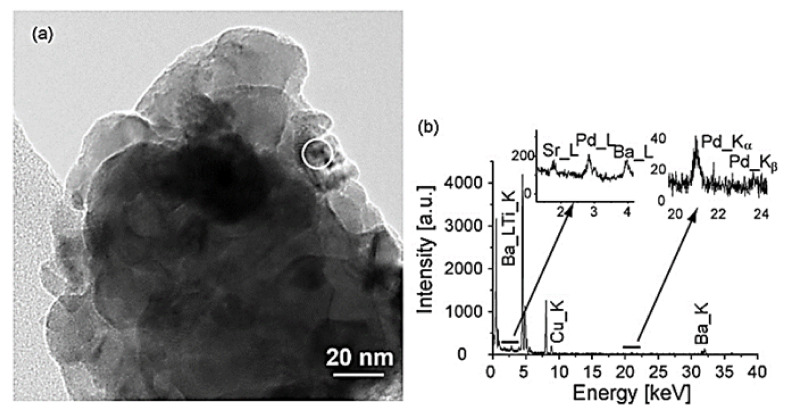
TEM of the BaTi_0.9_5Pd_0.05_O_3_ calcined up to 700 °C/3 h (in air): (**a**) a minute part of an agglomerate, (**b**) EDS spectrum of matrix area encircled in image a, regions containing the Pd–K and Pd–L X-ray diffractions are scaled up to provide a visible Pd-signal. Reproduced with permission from Ref. [85]. Copyright 2010, Elsevier.

**Figure 13:**
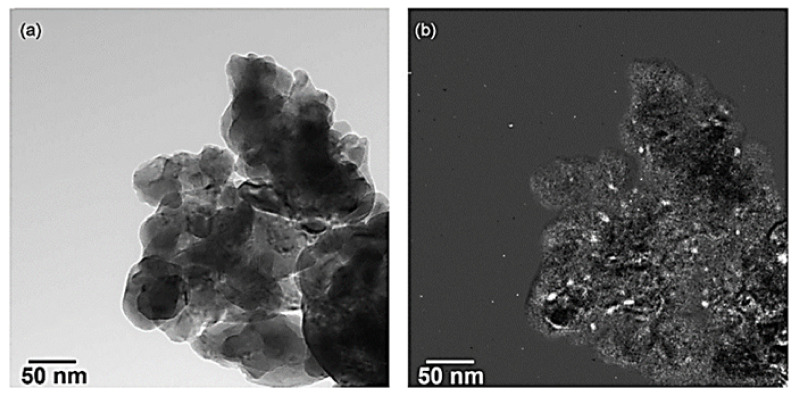
TEM of BaTi_0.95_Pd_0.05_O_3_ calcined up to 700 °C/3 h (in air), (**a**) image of a zero-loss filtered bright field (10 eV slit width), and (**b**) image of Pd elemental map using the 3-window method (i.e., 60 s/window, 20 eV slit width, slit centered at 315 eV, 325 eV, and 410 eV). Reproduced with permission from Ref. [85]. Copyright 2010, Elsevier.

**Figure 14:**
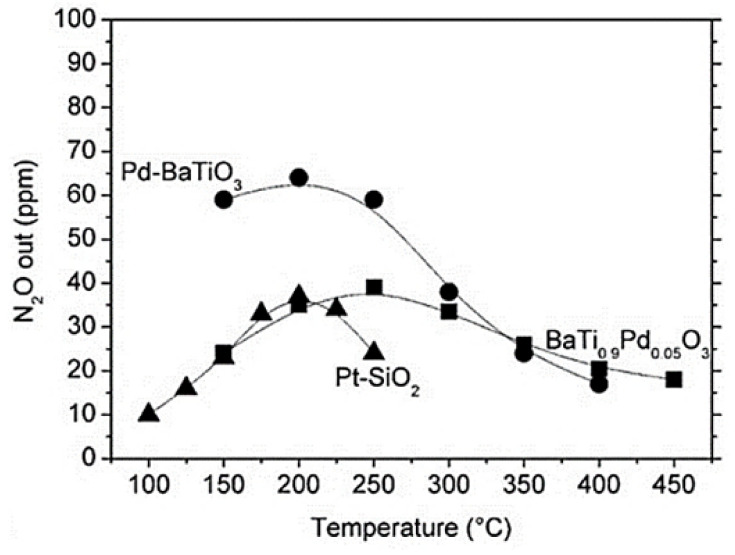
N_2_O formation of the catalysts (i.e., Pd-BaTiO_3_, Pt-SiO_2_, BaTi_0.95_Pd_0.05_O_3_). Reproduced with permission from Ref. [85]. Copyright 2010, Elsevier.

Mondragon Rodriquez et al. [85] have also prepared, via a co-precipitation method, two different perovskite-based catalysts, namely BaTi_0.95_Pd_0.05_O_3_ and Pd/BaTiO_3_, to evaluate their catalytic performance in the H_2_-SCR of NO_x_ reaction. Four experimental protocols were adopted in the study to decipher the effects of H_2_O, CO_2_, and CO co-feed, the WGHSV employed, as well as the calcination temperature of the materials during preparation. H_2_-TPR, XPS, SEM, TEM, and XRD measurements were adopted for the characterization of the materials under consideration. A TEM image of the BaTi_0.95_Pd_0.05_O_3_ perovskite calcined at 700 °C is shown in Figure 12a. Interestingly, a low Pd-content was found by the EDX-point measurements in the matrix (Figure 12a, arrow), while from Figure 12b qualitatively noticeable Pd-peaks were detected at various sites of the material. EF-TEM analysis was also applied to elucidate the presence of Pd particles before and after reduction pretreatment (Figure 12a and Figure 13b). That said, images using exclusively electrons of a specific energy loss were tracked via an imaging filter. In particular, Figure 13b showed the elemental distribution of Pd, which was calcined in air. Even though the reduction pretreatment was postponed in this sample, many Pd-containing particles were detected in the mapped image. On the other hand, from Figure 13a these particles were hardly visible, while their chemical nature was hardly identified. It was also reported that both Pd-containing perovskite phases and Pd-oxides may be present. Following exposure to the electron beam during the reduction of PdO metallic Pd can also be formed. Besides this vagueness, it was obvious that Pd-rich nanoparticles were present in the catalyst. The catalytic results showed that the use of a high calcination temperature deteriorates the deNO_x_ activity of the BaTi_0.95_Pd_0.05_O_3_ perovskite: the calcined at 500 °C BaTi_0.95_Pd_0.05_O_3_ catalyst exhibited higher NO conversion (ca. 90%) than that calcined at 900 °C, due to the lower surface area (53% lower) of the latter [85]. However, with respect to the N_2_ selectivity, no significant effects resulted. Under the use of a very high WGHSV (1.61 × 10^6^ mL∙g^−1^∙h^−1^), the authors reported maximum NO conversions of 50% at 250 °C and 70% at 200 °C for BaTi_0.95_Pd_0.05_O_3_ and Pd/BaTiO_3_ catalysts, respectively (Table 2). Considering the high space velocity of these catalytic tests, NO_x_ conversions were decent. In general, Pd/BaTiO_3_catalyst outperformed the BaTi_0.95_Pd_0.05_O_3_ catalyst in terms of catalytic activity below 250 °C, though more N_2_O was formed (Figure 14). On the other hand, above 250 °C the BaTi_0.95_Pd_0.05_O_3_ sample exhibited higher NO_x_ conversion compared to the Pd/BaTiO_3_ catalyst. It was also found [85] that the addition of CO in the feed afflicted the NO_x_ conversion, particularly in the temperature range of 160–195 °C. The authors argued that the negative effect of the presence of CO was smaller in comparison to the results presented in the literature. They also highlighted the oxidation potential of Pd which can promote the chemical adsorption of CO on Pd-containing surfaces below 200 °C. However, below this temperature, CO adsorption probably competed with NO_x_ reduction for occupying the same active sites. Above 200 °C, higher rates of CO oxidation were observed, though they still blocked the active sites of the catalyst compromising the NO_x_ reduction process. On this basis, small NO_x_ reduction levels were achieved in the presence of CO, while increased N_2_O formation rates were found between 160 and 195 °C. Finally, the conversion of NO_x_ was further decreased upon increasing the temperature while with respect to N_2_ selectivity a medium fluctuation was reported. Τhe appearance of two maxima of the NO_x_ conversion obtained between 150 and 200 °C during the NO_x_ reduction in the presence of CO was not interpretable from the available data. In the presence of CO_2_ and H_2_O, the catalyst maintained 58.7% of NO_x_ conversion for temperatures between 160 and 195 °C. Above 230 °C, maximum NO_x_ conversion (ca. 71.5%) was recorded, however, a further increase led to an inverse correlation between temperature and conversion. A beneficial effect on the catalytic performance of BaTi_0.95_Pd_0.05_O_3_/900 (calcined at 900 °C) was reported for temperatures between 195 and 270 °C, suggesting slightly decreased N_2_O formation in this temperature range. A positive effect by the addition of CO_2_ and H_2_O on NO conversion was also found for the BaTi_0.95_Pd_0.05_O_3_/900 catalyst. Nevertheless, below 200 °C, the presence of H_2_O resulted in a complex mechanism with antagonistic reactions, (i.e., N_2_O and N_2_ formation versus NO_x_ reduction), however above 200 °C the conversion of NO_x_ was improved. This behavior was attributed to the co-adsorption of CO_2_ and H_2_O during the NO_x_ reduction. Moreover, above this temperature H_2_O dissociation and H_2_O adsorption may be involved in the NO_x_ reduction mechanism [85].

In order to highlight the benefits that can be achieved for the H_2_-SCR of NO_x_ using perovskite-type catalysts, i.e., perovskites and/or perovskites promoted by very low noble metal (NM) loadings, in Table 2 we present comparative representative results obtained using perovskite-based catalysts and conventional supported noble metal catalysts. It can be concluded that perovskite-based catalysts are highly active and significantly selective toward N_2_ at temperatures typically below 200 °C; the achievements on these catalysts are well compared to those obtained using the more expensive NM-based conventional catalysts, typically containing higher NM loadings.

### 2.3. Perovskite Catalysts in CO-SCR of NO_x_

It is worth noting that our literature search on the perovskites-catalyzed selective (i.e., at excess O_2_ conditions) reduction of NO_x_ by CO was virtually fruitless as we managed to unearth only one publication [90]. On the contrary, and to our surprise, many reports were found on the reduction of NO by CO in the absence of O_2_. The former unexplained lack of interest can be partly understood by the fact that the management of CO as an externally supplied reducing agent (as is normally followed in the case of NH_3_−, HCs−, or H_2_−SCR processes) is difficult due to the extremely dangerous nature of this molecule. However, it should be noted that in many practical combustion processes CO is contained in a high concentration in the exhaust gases together with excess O_2_ [13,87,89]. Therefore, it would be of great interest to exploit its existence in the exhaust gases to reduce the coexisting NO_x_ (i.e., CO-SCR of NO_x_) regardless of the fact that its external supply is not desirable. In the following lines we first analyze the report that was found to be directly related to the CO-SCR of NO_x_ (i.e., the presence of excess O_2_) on perovskite-based catalysts, and then, due to the importance of the topic, we consciously choose to deviate slightly from the title of the present review by analyzing the literature on reduction of NO by CO over perovskites even in the absence of O_2_.

**Figure 15:**
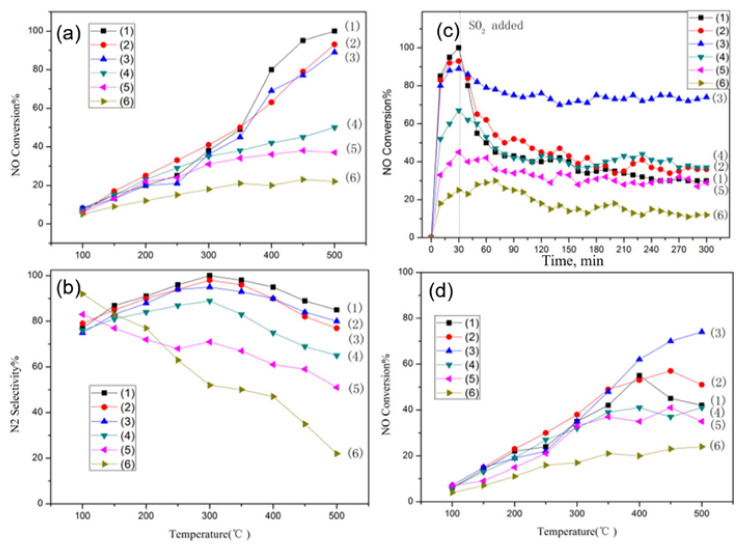
NO conversion over La_x_Ce_1__−x_FeO_3_ perovskites during the NO reduction by CO: NO conversion versus temperature from 100 to 500 °C (**a**), and corresponding N_2_-selectivities (**b**) using 400 ppm NO/500 ppm CO/He balance at 1 bar and GHSV of 24,000 h^−1^; NO conversion versus time of La_x_Ce_1__−x_FeO_3_ catalysts at 500 °C with 100 ppm SO_2_ in the feed (**c**); NO conversion versus temperature of La_x_Ce_1__−x_FeO_3_ catalysts in CO + NO reaction with the addition of 100 ppm SO_2_, 3% O_2_ and 3 vol.% H_2_O in the feed (**d**). (1): LaFeO_3_, (2): La_0.8_Ce_0.2_FeO_3_, (3): La_0.6_Ce_0.4_FeO_3_, (4): La_0.4_Ce_0.6_FeO_3_, (5): La_0.2_Ce_0.8_FeO_3_, and (6): CeO_2_. Reproduced with permission from Ref. [90]. Copyright 2016, Elsevier.

Qin et al. [90] investigated the effect of ceria-content in La_x_Ce_1-x_FeO_3_ (x = 0.2, 0.4, 0.6, 0.8, 1) perovskite catalysts on improving SO_2_ resistance for catalytic reduction of NO with CO in the presence of O_2_. Specifically, the feed gas was comprised of 400 ppm NO/500 ppm CO/3% O_2_/3% H_2_O/(100 ppm SO_2_) balance N_2_ at a GHSV of 24,000 h^−1^, and the temperature range of catalytic tests was 100–500 °C. The perovskites were characterized by SO_2_−TPD, CO−TPR, XRD, and XPS measurements. In the absence of H_2_O, O_2_, and SO_2_ in the feed (NO + CO reaction) results suggested better catalytic performance with respect to both NO conversion (Figure 15a) and N_2_− selectivity (Figure 15b) of LaFeO_3_ catalyst. To investigate the effect of SO_2_ in the NO conversion activity of the perovskites 100 ppm of SO_2_ was added into the NO + CO feed after 30 min of operation and the transient experiments were kept running for a total of 300 min time-on-stream (Figure 15c). Obviously, the existence of SO_2_ inhibits the catalytic activity of all perovskites but to a different degree. La_0.6_Ce_0.4_FeO_3_ was less inhibited in the presence of SO_2_ highlighting the beneficial effect of substituting La by Ce in the A-sites of the perovskite on its resistance to SO_2_-poisoning. It is also apparent that exceeding La substitution by Ce in the perovskite is detrimental to both activity (Figure 14a) and SO_2_-poisoning resistance (Figure 15c). The authors also evaluated the materials at CO-SCR conditions, i.e., in the presence of excess O_2_, and co-presence of H_2_O, and SO_2_ (Figure 15d). The La_0.6_Ce_0.4_FeO_3_ perovskite which was found to be optimal in the previous sets of experiments was again optimal in this case in particular at high temperature, however, no results for nitrogen-containing products are available in order to see its N_2_-selectivity behavior as well; actually, at high temperatures, the NO_2_ production via the NO + O_2_ reaction is favored. Based on their characterization results, the authors conclude that the optimal La_0.6_Ce_0.4_FeO_3_ catalyst retains its original structure during the reaction, and the specific redox properties of Ce in its structure are the cause of its superior activity and SO_2_ tolerance performance [90].

We now turn our attention to works that report on the performance of perovskite materials during the reduction of NO by CO in the absence of O_2_. Wu et al. [91] employed a sol–gel method to prepare LaM_0.5_Mn_0.5_O_3_ (M = Cu, Co, Fe, Ni, Cr) perovskite catalysts in order to evaluate the effect of partial substitution on B-site of this series of catalysts in NO reduction by CO in the absence of O_2_ but using excess H_2_O (10%) in the feed: 10% CO/5% NO/He balance (i.e., excess CO) and occasionally 10% H_2_O and 1000 ppm SO_2_. A variety of WGHSVs ranging from 60,000 to 600,000 mL∙g^−1^∙h^−1^ were employed, and 72 h time-on-stream stability tests at 250 °C were also conducted. The study includes materials characterization by XPS, ICP-AES, O_2_-TPD, H_2_-TPR, XRD, BET, and in situ DRIFTS measurements. It was demonstrated that the NO reduction performance of the perovskites clearly improved via the partial substitution of the B-sites by the aforementioned elements, following the order LaCu_0.5_Mn_0.5_O_3_ > LaCr_0.5_Mn_0.5_O_3_ > LaNi_0.5_Mn_0.5_O_3_ > LaCo_0.5_Mn_0.5_O_3_ ≈ LaFe_0.5_Mn_0.5_O_3_ > LaMnO_3_. LaCu_0.5_Mn_0.5_O_3_ catalyst displayed the best catalytic behavior with complete removal of NO at 300 °C and 100% selectivity toward N_2_. On the other hand, the bare LaMnO_3_ displayed the worst behavior in terms of both NO conversion and N_2_-selectivity compared to other Cu-modified perovskites. The authors also examined the stability of the catalysts by carrying out 72-h time-on-stream stability tests at 750 °C. Results indicated that the NO conversion of LaMnO_3_ dropped from 22.2 to 10.3% whereas the NO conversion of LaCu_0.5_Mn_0.5_O_3_ was barely affected (i.e., 63.8 to 59.3%). The recycle catalytic capacity for 10 cycles was also conducted using the bare LaMnO_3_ and the optimal LaCu_0.5_Mn_0.5_O_3_ catalysts. A gradual decrease of the catalytic performance was reported for both catalysts, however, the degree of degradation was less significant for the Cu-containing catalyst, highlighting once again the positive role of Cu addition. XRD supportive measurements during these experiments also corroborated the superiority of LaCu_0.5_Mn_0.5_O_3_ over LaMnO_3_, by showing that the crystallinity of LaCu_0.5_Mn_0.5_O_3_ was less affected (i.e., Cu addition hindered the decrease of crystallinity in the bulk phase) in comparison to that of the bulk phase during the cycles. Moreover, XPS analysis illustrated the existence of constant electron binding energy for the Cu-containing sample during the cycles, indicating an invariant valence state of Mn and La species under the reaction conditions [91]. On the other hand, it was also clear that the LaMnO_3_ sample was less resistant to the reaction atmosphere. The effect of WGHSV (i.e., 60,000–600,000 mL∙g^−1^∙h^−1^) was also studied for these two catalysts showing a gradual shift of the NO conversion profiles to higher temperatures upon increasing space velocity for both catalysts.

**Figure 16:**
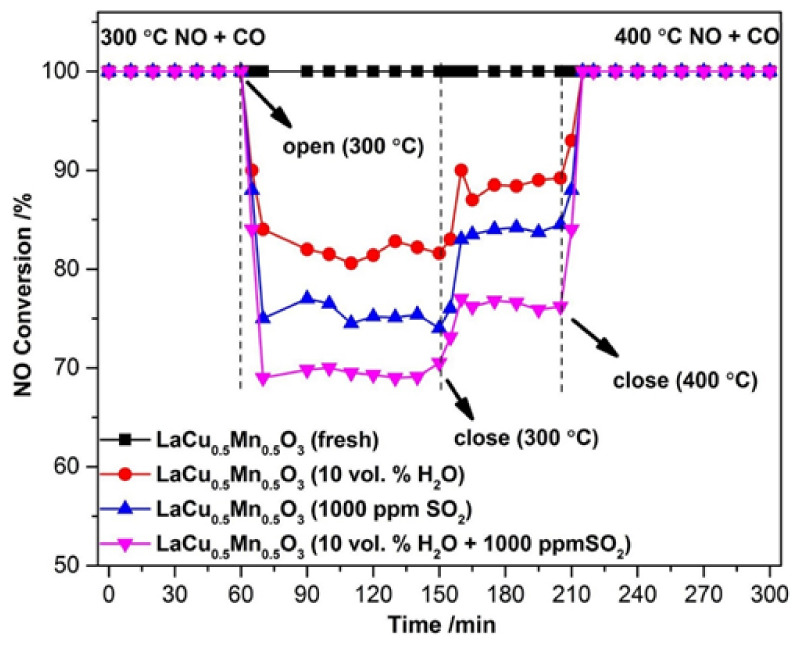
The NO conversions over LaCu_0.5_Mn_0.5_O_3_ catalyst upon adding different reaction inhibiting substances (10% H_2_O/1000 ppm SO_2_ and 10 % H_2_O + 1000 ppm SO_2_) in the NO + CO reactor feed stream. Reproduced with permission from Ref. [91]. Copyright 2020, Elsevier.

**Figure 17:**
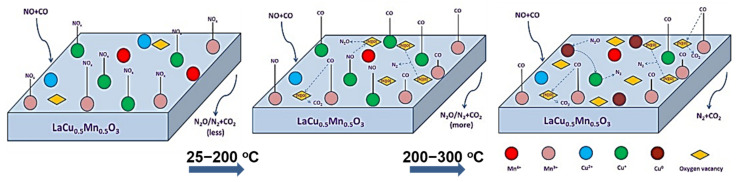
The mechanism of catalytic reduction NO by CO on LaCu_0.5_Mn_0.5_O_3_ perovskites. Reproduced with permission from Ref. [91]. Copyright 2020, Elsevier.

The sulfur and H_2_O resistance capacity of the optimal LaCu_0.5_Mn_0.5_O_3_ catalyst was also probed during time-on-stream experiments at a constant temperature in which 10% H_2_O or 1000 ppm SO_2_ or even 10% H_2_O + 1000 ppm SO_2_ was co-fed with the flowing NO + CO gas mixture (Figure 16) [91]. As can be seen, the addition of H_2_O led to an about 16% drop in NO conversion which then maintained approximately constant. When H_2_O was removed from the feed, the catalytic performance was only partially restored. The effects of inhibition/recovery on catalytic behavior due to the introduction/removal of SO_2_ into the feed stream were qualitatively similar to those of H_2_O but more pronounced, and become even more intense when both SO_2_ and H_2_O are fed (Figure 16). An increase of the temperature from 300 to 400 °C was needed to lead to a complete restoration of catalytic activity after removal of the inhibitors from the feed stream, regardless of their identity. That said, this Cu-containing perovskite catalyst could be considered a fairly good sulfur and H_2_O resistant material. In situ DRIFTS experiments enabled the authors to suggest an Eley–Rideal reaction mechanism (Figure 17) which adequately describes their findings. As shown in Figure 17, introducing NO and CO at ambient temperature can lead to the attachment of NO molecules onto the perovskite, covering the active sites indicated in the figure. Therefore, some nitrates and nitrate-like species may be formed on the perovskite’s surface inhibiting the adsorption of CO molecules. Below 200 °C, the adsorbed NO species may react with CO to form N_2_, N_2_O, and CO_2_, as corroborated by both in situ DRIFTS measurements and catalytic runs under a real reaction atmosphere. Above 200 °C, the desorption, dissociation, and conversion of NO species can be observed to form N_2_O and N_2_ (i.e., NO → [N] + [O], NO + [N] → N_2_O, [N] + [N] → N_2_).

**Figure 18:**
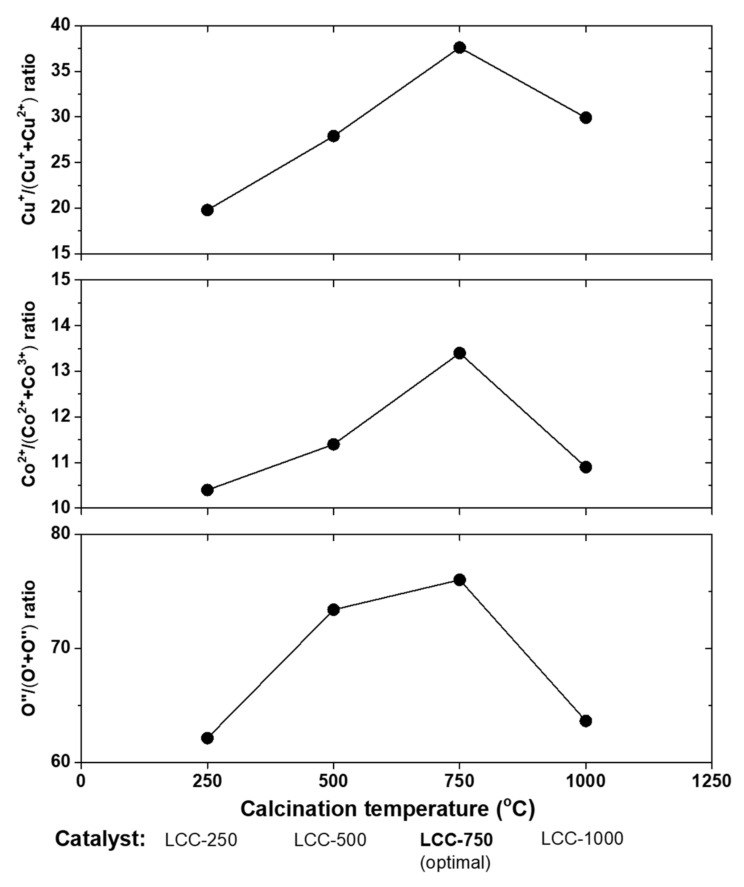
The surface element distribution of LCC-x (x = 250, 500, 750 and 1000 °C) perovskites calcined at different temperatures. O′ and O″ are bulk and chemically absorbed oxygen, respectively; O′ is connected with the redox capacity of the catalysts, and the proportion of O″ is closely linked to the quantity of oxygen deficiency in catalysts. Data were taken with permission from Ref. [92]. Copyright 2019, Elsevier.

In a different study, using the same preparation method (sol–gel) the research group also prepared a LaCu_0.25_Co_0.75_O_3_ (LCC) perovskite to study the effect of calcination temperature (250, 500, 750, and 1000 °C) on its catalytic performance for NO + CO reaction tested in a feed composition of 10% CO/5% NO/Ar balance at WGHSV = 60,000 mL∙g^−1^∙h^−1^ and temperature range between 100–600 °C [92]. Regarding the NO and CO conversion efficiency, the LCC-750 > LCC-500 > LCC-250 > LCC-1000 sequence was found, with the outperformed LCC-750 sample to achieve complete NO and 50% CO conversions at ~350 °C, and 100% selectivity toward N_2_ at ~400 °C. The authors concluded that the ratio of Cu^+^/(Cu^+^ + Cu^2+^) and Co^2+^/(Co^2+^ + Co^3+^), the reducibility, and amount of oxygen deficiencies were the key points for the enhancement catalytic behavior observed. Indeed, maximum values of all these ratios and properties were measured on the outperformed LCR-750 catalyst (Figure 18).

In yet another study, the same group reported the effect of B-site partial substitution of a La_0.8_Ce_0.2_M_0.25_Co_0.75_O_3_ (M = Fe, Mn, Cu) perovskite on NO reduction by CO [93]. The preparation method and reaction conditions used were similar as above. It was found that the addition of all Fe, Mn, and Cu on the bare La_0.8_Ce_0.2_CoO_3_ perovskite was beneficial to its NO reduction activity, however, the Cu-substituted La_0.8_Ce_0.2_Cu_0.25_Co_0.75_O_3_ perovskite outperformed all the other samples on both activity and N_2_ selectivity. In terms of maximum achievements, the Cu-substituted perovskite showed 100% NO conversion, 50% CO conversion, and 99% N_2_ selectivity at ~300 °C. This catalyst has also been found to be substantially more stable in 48-h time-on-stream performance; a slight (82% → 80.3%) compared to a significant (58.8% → 43.6%) degradation of NO conversion activity was recorded for La_0.8_Ce_0.2_Cu_0.25_Co_0.75_O_3_ and La_0.8_Ce_0.2_CoO_3_, respectively. The performance superiority of La_0.8_Ce_0.2_Cu_0.25_Co_0.75_O_3_ was attributed to the enhanced amount of O_2_ vacancies, texture properties, and reducibility, while the NO + CO reaction kinetics appeared to comply with the Eley–Rideal mechanism [93].

**Figure 19:**
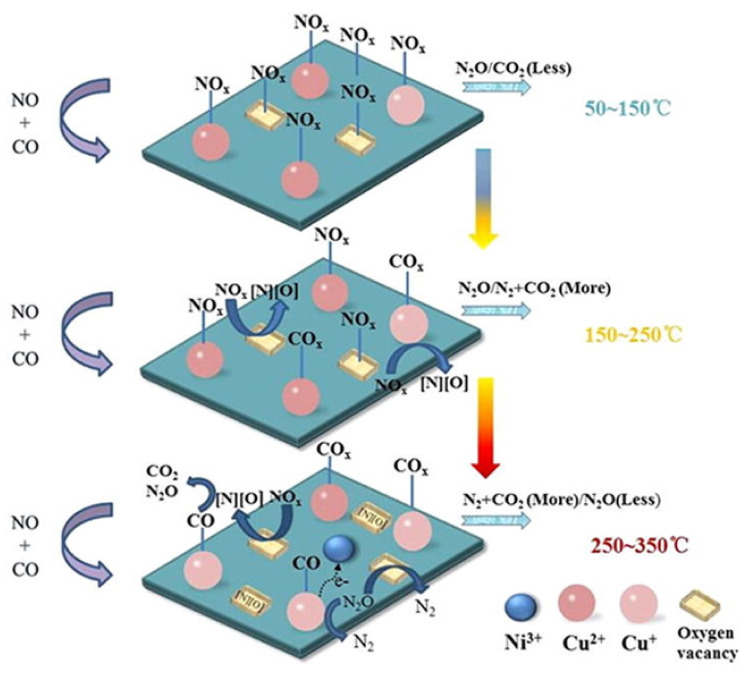
Reaction mechanism for the catalytic reduction of NO by CO on LaNi_0.5_Cu_0.5_O_3_ perovskites. Reproduced with permission from Ref. [94]. Copyright 2019, Elsevier.

Moreover, the same group also studied a series of LaNi_0.5_M_0.5_O_3_ (M = Co, Mn, Cu) perovskites using again a NO + CO feed with excess CO (i.e., 5% NO/10% CO/85% He at WGHSV = 36,000 mL∙g^−1^∙h^−^^1^) [94]. Results illustrated that the partial (50 mol%) substitution of Ni with Co, Mn, or Cu in LaNiO_3_ had in all cases a positive effect on its catalytic activity, with Cu-substituted LaNi_0.5_Cu_0.5_O_3_ perovskite outperforming all others in N_2_ selectivity at temperatures > 250 °C. In situ DRIFTS experiments enabled the authors to propose a Langmuir–Hinshelwood mechanism for the NO reduction by CO on this series of perovskites as schematically shown in Figure 19, exemplified for LaNi_0.5_Cu_0.5_O_3_ perovskite [94]. According to the mechanism, at low temperatures (50–150 °C) NO is preferentially adsorbed on the active sites of the perovskite surface forming nitrates, which can be gradually desorbed and react with gaseous CO forming small amounts of N_2_O, N_2_, and CO_2_. In the temperature region 150–250 °C, Cu^2+^ is reduced to Cu^+^ providing sites for CO chemisorption that produce some carbonate and carboxylate species. At the same time, surface oxygen vacancies can activate NO dissociative adsorption and facilitate N_2_O decomposition. The as derived dissociative products can further react with adsorbed CO forming CO_2_ and N_2_O. For high temperatures (ca. 250 °C and higher) chemisorbed O species began to desorb regenerating oxygen vacancy sites. The increased availability of the latter sites further facilitates NO and N_2_O dissociation. Then Cu^2+^ sites can be regenerated via Ni^3+^ + Cu^+^ ↔ Ni^2+^ + Cu^2+^, and Cu^+^ also can be oxidized by N_2_O to Cu^2+^ leading to N_2_O conversion toward N_2_ (Figure 19) [94].

Tarjomannejad et al. [95] prepared via a sol–gel method LaMn_1-x_Fe_x_O_3_ (x = 0, 0.3, 0.5, 0.7, 1) and La_0.8_M_0.2_Mn_0.3_Fe_0.7_O_3_ (M = Ce, Ba, Cs, Sr) perovskites to evaluate their performance in catalytic reduction of NO by CO under a 3000 ppm NO/3000 ppm CO/Ar balance gas feed at a WGHSV = 12,000 mL∙g^−1^∙h^−1^ in the temperature range between 150 and 500 °C. The catalysts under consideration were characterized using SEM, H_2_-TPR, XPS, BET, and XRD techniques. Results showed that the LaMn_0.3_Fe_0.7_O_3_ catalyst exhibited the highest catalytic activity when compared to the other catalysts of the LaMn_1-x_Fe_x_O_3_ group. In particular, the catalytic activity of this group followed the order LaMnO_3_ < LaFeO_3_ < LaMn_0.7_Fe_0.3_O_3_ < LaMn_0.5_Fe_0.5_O_3_ < LaMn_0.3_Fe_0.7_O_3_. It was reported that the partial substitution of Mn by Fe in the perovskite led to the formation of O_2_ vacancies promoting the catalyst’s reducibility. The introduction of a small amount of Ce, Sr, Cs into the A-site of the perovskite had a beneficial effect on catalytic performance. In contrast, partial substitution of La by Ba compromised the catalyst activity. On this basis the catalytic activity followed the order La_0.8_Ba_0.2_Mn_0.3_Fe_0.7_O_3_ < LaMn_0.3_Fe_0.7_O_3_ < La_0.8_Cs_0.2_Mn_0.3_O_3_ < La_0.8_Sr_0.2_Mn_0.3_Fe_0.7_O_3_ < La_0.8_Ce_0.2_Mn_0.3_Fe_0.7_O_3_. The authors concluded that the enhanced activity of the best and second-best catalyst (obtained by substitution of La by Ce^4+^ and Sr^2+^ in the A-site) was due to the induced chances in the reducibility of B-site cations, Mn^4+^/Mn^3+^, and Fe^4+^/Fe^3+^ ratios, and increase the O_ads_/O_latt_ ratio, factors that increase the number of structural defects in the perovskite structure. In an additional publication of the research group [35], LaCu_0.7_B_0.3_O_3_ (B = Mn, Fe, Co) perovskites were synthesized and comparatively evaluated in NO + CO reaction at feed conditions such as the above. After finding the superiority of LaCu_0.7_Mn_0.3_O_3_, they further modified the A-sites of this perovskite by an alkali or alkaline earth metal (Rb, Sr, Cs, Ba) and concluded that La_0.8_Sr_0.2_Cu_0.7_Mn_0.3_O_3_ was the best among all samples tested, offering 100% NO conversion at 375 °C. The same arguments as in the previous study were invoked to explain their findings.

The same group [96] prepared two groups of perovskites, LaFe_0.5_M_0.5_O_3_ and LaMn_0.5_M_0.5_O_3_ (M = Cu, Co, Mn, Fe), and evaluated them in NO + CO reaction using stoichiometric conditions (3000 ppm NO/3000 ppm CO/Ar balance at WGHSV = 12,000 mL∙g^−1^∙h^−1^ and temperatures between 100 and 450 °C). The catalytic activity for the LaFe_0.5_M_0.5_O_3_ group followed the order LaFeCo < LaFe < LaFeCu < LaFeMn. The catalytic activity for the LaMn_0.5_M_0.5_O_3_ group followed the order LaMn < LaMnCo < LaMnFe < LaMnCu. Among all the samples tested (both series) the optimal behavior overall was that of LaMn_0.5_Cu_0.5_O_3_. It was associated with a synergistic interaction between Mn and Cu, higher reducibility at low temperatures, and an increased number of structural defects. The authors also examined three different mechanisms for the NO reduction by CO. Among them, the Langmuir–Hinshelwood model was found to be more suitable for describing the kinetic data obtained. More specifically, the proposed mechanism is that described by the following reaction steps (R.1)–(R.6), which is similar to that reported for noble metal catalyzed NO + CO reaction [97,98].

NO + * ↔ NO*(R.1)

CO + * ↔ CO*(R.2)

NO* + * → N* + O*(R.3)

N* + N* → N_2_ + 2* (R.4)

N* + NO* → N_2_O + 2* (R.5)

CO* + O* → CO_2_ + 2* (R.6)

**Figure 20:**
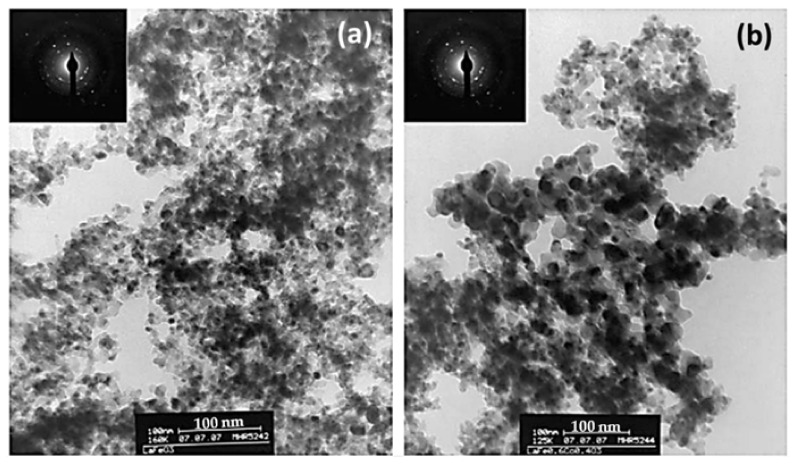
TEM images of LaFeO_3_ (**a**) and LaFe_0.6_Co_0.4_O_3_ (**b**) prepared by a nanocast method. Reproduced with permission from Ref. [99]. Copyright 2009, Elsevier.

**Figure 21:**
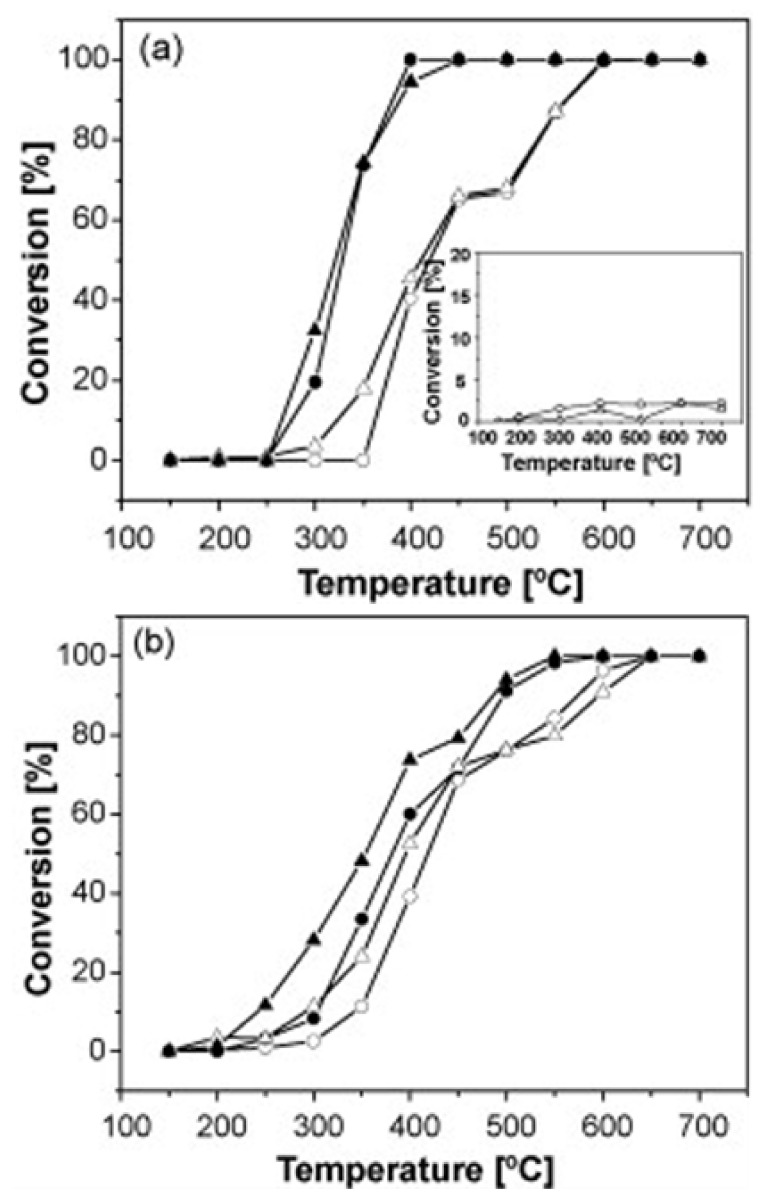
Temperature profiles for the conversion of NO to N_2_ (○, ●) and CO to CO_2_ (▵, ▴) on: (**a**) uncast LaFeO_3_ (○, ▵) and nanocast LaFeO_3_ (●, ▴). Inset: conversion of NO to N_2_ (○) and CO to CO_2_ (▵) without any catalyst; (**b**) uncast LaFe_0.6_Co_0.4_O_3_ (○, ▵) and nanocast LaFe_0.6_Co_0.4_O_3_ (●, ▴). Reproduced with permission from Ref. [99]. Copyright 2009, Elsevier.

De Lima et al. [99] prepared LaFe_1−*x*_Co*_x_*O_3_ perovskites, namely LaFeO_3_ and LaFe_0.6_Co_0.4_O_3_, synthesized either conventionally (by the citrate method) or using a nanocasting method; the latter leads to materials constituted by more than 97 wt% of perovskite phase and by agglomerates smaller than 100 nm constituted by crystallites of about 6 nm (Figure 20). As a result, the nanocast perovskites had about 10 times larger specific surface areas compared to the conventional perovskites (e.g., 49.3 and 30.5 vs. 5.6 and 3.6 m^2^/g for the nanocast and conventional LaFeO_3_ and LaFe_0.6_Co_0.4_O_3_ perovskites, respectively). These materials were comparatively evaluated in the reduction of NO by CO. Figure 21 shows the results of this comparison. Obviously, the nanocast perovskites are significantly more active than their conventional counterparts, and as the authors conclude this is mainly due to the higher specific surface area of the former and the consequent higher number of accessible active sites exposed to the reactants.

**Figure 22:**
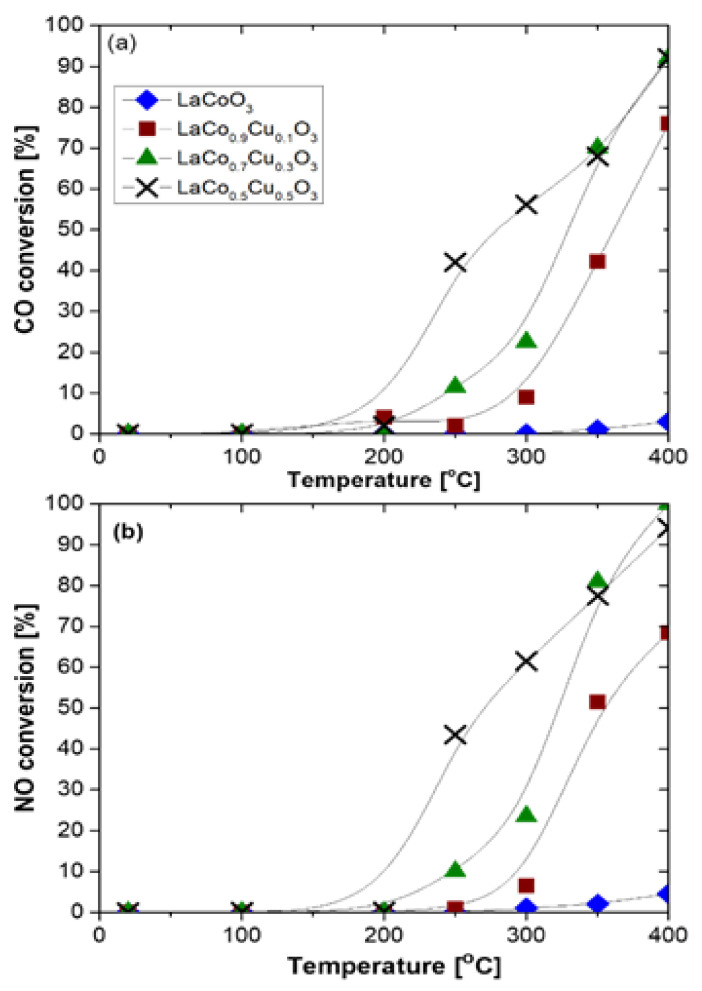
CO (**a**) and NO (**b**) conversion as a function of temperature on LaCo_0.5_Cu_0.5_O_3_, LaCo_0.7_Cu_0.3_O_3_, LaCo_0.9_Cu_0.1_O_3_, and LaCoO_3_ perovskites. Conditions: 4% NO/4% CO/balance He at 1 bar; WGHSV = 150,000 mL∙g^−1^∙h^−1^. Reproduced with permission from Ref. [59]. Copyright 2016, Elsevier.

Finally, aiming at three-way catalysis (TWC), Glisenti et al. [59] prepared largely Cu-doped LaCo_1−x_Cu_x_O_3_ (x = 0, 0.1, 0.3, and 0.5) perovskites by means of the citrate method and tested these materials in model reactions involved in TWC (i.e., NO + CO and CO + O_2_) as well as at simulated automotive exhaust conditions. Regarding the NO + CO model reaction the feed gas composition comprised of 4% CO/4% NO/Ar balance at 1 bar with a WGHSV = 150,000 mL∙g^−1^∙h^−1^. The catalysts under consideration were characterized by a variety of techniques. XRD results corroborated for a stable perovskite phase holding a rhombohedral geometry with the crystallite size to be decreased upon increasing the copper amount. XPS results suggested that the addition of Cu caused the decrease of Co(III) → Co(II) → Co(0) reduction temperatures, as a result of H_2_ activation. This H_2_ activation can be assigned to the surface segregated Cu clusters and to the increased O_2_ mobility because of the formation of vacancies. Regarding the catalytic performance of the materials in NO reduction by CO, it has been shown that the introduction of Cu into the LaCoO_3_ structure was beneficial as almost complete NO and CO conversions were achieved at 400 °C. Specifically, the catalytic activity of the samples followed the order LaCoO_3_ < LaCo_0.9_Cu_0.1_O_3_ < LaCo_0.7_Cu_0.3_O_3_ < LaCo_0.5_Cu_0.5_O_3_. That is the perovskite with the highest Cu doping outperformed the other samples (Figure 22); notably, N_2_ was the main N-containing reaction product [59].

As in the previous sections, we constructed Table 3 here, comparing the aforementioned results (and some additional ones) from the literature on NO reduction by CO using perovskite catalysts, even though, as mentioned at the beginning of the section, literature on the selective catalytic reduction (i.e., in excess of O_2_) of NO_x_ by CO is rather rare. For comparison, the table also includes some representative results on the titled reaction catalyzed by NM-based catalysts.

## 3. General Outcomes and Future Perspectives

This work reviews the literature that concerns the use of perovskites and perovskites-based catalysts in the selective catalytic reduction (SCR) of NO_x_ with different than the typically used NH_3_ or urea reducing agents, i.e., C_x_H_y_/C_x_H_y_O_z_, H_2_, and CO. The main purpose of this undertaking is to present the state-of-the-art in the field, which could help industry and academia become aware of new possibilities and perspectives in order to meet future requirements in addressing NO_x_ emissions.

The urgency of developing efficient approaches for NO_x_ emission control in order to meet increasingly stringent environmental requirements is widely accepted. Significant downsides are associated with low NO_x_ concentration and excess oxygen in the exhaust gas, conditions under which the competitive role of dioxygen in the oxidation of reducing agents to the detriment of NO_x_ reducing reactions to harmless N_2_ is major. During SCR of NO_x_, several catalysts and conditions can also favor N_2_O production which has undesirable high global warming potential and is also the main current cause of ozone depletion in the stratosphere. To this date N_2_O emissions are not actually regulated by the EU, however, this matter will definitely be of future interest. That said the development of selective catalytic systems to reduce NO_x_ emissions while using low-cost, non-noble metal-containing, catalytic systems is of great importance. Perovskite-based catalysts appear to be potential candidates for this purpose.

The low cost of perovskites combined with their unique physicochemical properties (i.e., redox/mixed valency properties of metals, O_2_-mobility, and bulk and surface oxygen vacancies) and versatility of composition render this class of materials as great candidates for SCR of NO_x_.

The easiness of substituting A and B-sites in ABO_3_ and A_2_BO_4_ perovskite formulas with rare earth metals, alkali, or alkaline earth elements (at A-sites), and transition metals from the 3d, 4d, or 5d configuration (at B-sites), endows them with a variety of different active sites capable of facilitating the adsorption of the reactants on different sites, thus eliminating the catalytic rate inhibitory competitive adsorption of reactants on the same sites.

The incorporation of noble metals such as Pd into the B-site seems an interesting option to enhance catalytic performance since there is an increase in both cationic defects and lattice oxygen mobility. In addition, using perovskites as supporting materials in low NM-loading supported catalysts can provide benefits through metal-support interactions corroborated by the high reducibility and oxygen ion mobility and capacity characteristics of perovskites.

Several papers have been published on perovskite-catalyzed C_x_H_y_/C_x_H_y_O_z_-, H_2_-, and CO-SCR of NO_x_. Most of these involved H_2_-SCR, a few C_x_H_y_/C_x_H_y_O_z_-SCR, and almost none CO-SCR. Representative cases of these works were analyzed in detail in this review and the achievements were presented comparatively in three comprehensive tables at the end of each respective section. For the sake of further convenience of comparison, the tables also include some results from NM-based catalysts applied under similar SCR conditions.

General conclusions that can be drawn and the corresponding perspectives are as follows:

- The SCR of NO_x_ behavior of perovskites (both in activity and N_2_-selectivity) is comparable, if not better (especially at low temperatures) to that of NM-based catalysts. Their time-on-stream stability and SO_2_ tolerance are also remarkable.
- Τhe partial replacement of A and/or B-sites with other suitable elements allows a significant improvement and controlled optimization of their SCR performance. For example, partially substituted with Cu perovskites were found to be significantly more active in comparison with the bare sample, due to the additional effect of the advantageous in catalysis Cu^2+^/Cu^+^ redox couple.
- Preparation methods capable of providing perovskites with a larger specific surface area are particularly advantageous due to the increased number of accessible active sites exposed to the reaction mixture. The typical specific surface of perovskites produced by traditional methods ranges between 5 and 20 m^2^∙g^−1^. Advanced or modified classical methods have been reported which can raise these values by two or even three times resulting in a significant catalytic benefit during SCR of NO_x_. Currently, significant efforts have been put to synthesize perovskites with surface areas as high as 100 m^2^∙g^−^^1^.
- Extensive characterizations of the synthesized perovskite materials that were frequently applied allowed the researchers to better understand the reaction pathways, the nature, and the role of the active sites, thus extracting relatively reliable morphology–activity correlations. However, apart from the in situ DRIFTS studies, no other operando techniques such as in situ XRD and in situ TEM were found to have been applied to the studies included herein. In light of such shortcomings, the frequent borrowing of reaction mechanisms from those proposed for analogous NM-based systems is justified. However, the use of perovskites in the SCR process is more likely to introduce new, easier reaction pathways that need to be in-depth understood in order to proceed with a coordinated optimization of perovskite composition for the SCR of NO_x_. At the same time, DFT calculations that are generally missing from the documents included herein can be particularly helpful in the above objectives.
- On the other hand, modern approaches in catalysis have emerged following the pioneering work of the Hamada team [50] on what is now described as “*exsolution*” that offers new perspectives on the use of perovskites. The creation of different kinds of alloy or metal particles at nano or even atomic sizes on the surface of perovskites may provide the chance of tailoring the local surface properties and metal–support interactions, leading to enhanced performance. The active interfaces generated by the exsolution process can also result in higher activity and stability for this type of catalyst. The method could provide effective solutions in the field of SCR of NO_x_ as well. Due to the recency of the discovery, applications focused on the specific topic of this review have not yet been found (the work of Hamada and co-workers was implemented in TWC conditions). We could assume that the *exsolution* concept will be an intense research approach in the coming years on NO_x_ abatement under lean conditions.

It is obvious from the above that there is significant free space and many degrees of freedom for object research, which can bring significant environmental benefits and value. Perovskites as materials show unique handling properties and tailoring them through their composition could optimize SCR of NO_x_. They are thermally stable, rather tolerant of poisons, and above all seem to work adequately with all possible reducing agents considered herein.

The specialized properties of perovskites, such as multiple types of active centers including surface oxygen vacancies, as well as labile lattice oxygen and mobile O^2−^ ions are particularly useful in catalysis. According to recent discoveries, these properties can play multiple roles as reaction promoters and as stabilizers of dispersed catalyst nanoparticles providing catalysts with high anti-sintering characteristics [26,27,28,29,30].

Even though the total exclusion of precious metals in catalytic processes seems unrealistic in the short run, combined use of both may provide substantial technological advantages. Based on the significant research carried out in this field, it can be argued that perovskites can serve as suitable active supports for precious metals, allowing both the reduction of noble metal loadings and the extension of their lifetime, bearing in mind the issues mentioned in the above paragraph.

As has been shown, the deNO_x_ efficacy of perovskites is remarkable for all the reducing agents considered herein. This provides additional practical benefits and alternatives. We believe that the involvement of perovskites in SCR of NO_x_ could further expand the Environmental Catalysis Society’s potential for a cleaner environment, although catalysis has virtually unlimited outlets. More efforts are needed in fundamental and application studies for deNO_x_ processes catalyzed by perovskites to fully enhance their potential in the field—there is ample open space for promising research and development on the subject.

## Figures and Tables

**Table 1:** Perovskite-catalyzed H_x_C_y_(O_z_)-SCR of NO_x_ representative studies.

Catalyst	Reaction Feed Conditions					Achievements			Ref.
	NO (%)	HC (%)	O_2_ (%)	Other (%)	WGHSV (mL∙g^−1^∙h^−1^)	X_NO_ (%)	at T (°C)	S_N2_ (%)	
**Perovskite and NM/perovskite catalysts**									
**LaFe_0.8_Cu_0.2_O_3_-RG** **LaFe_0.8_Cu_0.2_O_3_-CA** **LaFeO_3_-CA**	0.1 0.1 0.1	0.3 (CH_3_OH) 0.3 (CH_3_OH) 0.3 (CH_3_OH)	8 8 8	- - -	30,000 30,000 30,000	>90 >90 >80	>430 >475 >575	n/a n/a n/a	[74] [74] [74]
**La_0.8_Sr_0.2_MnO_3_/α-Al_2_O_3_** **La_0.8_Sr_0.2_MnO_3_/α-Al_2_O_3_**	0.1 0.1	0.12 (CH_4_) 0.12 (CH_4_)	0 5	- -	1636 h^−1^ (GHSV) 1636 h^−1^ (GHSV)	>90 96	>875 800	n/a n/a	[76] [76]
**La_0.8_Sr_0.2_MnO_3_**	0.4	0.24 (C_10_H_22_)	9	1.5 (H_2_O)	36,000	20–65	200–275	13 (max at 210 °C)	[77]
**Conventional, supported on oxide supports NM catalysts**									
**2wt%Pt/SiO_2_**	0.4	0.24 (C_10_H_22_)	9	1.5 (H_2_O)	36,000	>90	200–250	18 (max at 200 ^o^C)	[77]
**0.5wt%Pt/** **γ** **-Al_2_O_3_** **0.5wt%Pt(1.6wt%Na)/** **γ** **-Al_2_O_3_**	0.1 0.1	0.1 (C_3_H_6_) 0.1 (C_3_H_6_)	5 5	- -	180,000 * 53,485 *	>50 >50	300–400 225–375	40 (at 300 °C) 75 (at 225 °C)	[7] [7]

* The same contact time of the reactants with the catalyst active sites was imposed in these two cases by adjusting WGHSV.

**Table 2:** Comparative presentation of the achievements obtained for H_2_-SCR of NO_x_ by using representative perovskite-based catalysts and conventional-type noble metal catalysts.

Catalyst	Reaction Feed Conditions					Achievements			Ref.
	NO (%)	H_2_ (%)	O_2_ (%)	Other (%)	WGHSV (mL∙g^−1^∙h^−1^)	X_NO_ (%)	at T (°C)	max. S_N2_ (%)	
Perovskite and NM/perovskite catalysts									
LaFe_0.65_Co_0.3_Pd_0.05_O_3_	0.072	1	5	7.2 (H_2_O) + 7.2 (CO_2_)	55,400	>50 (max. 57)	200–250	75 (at 200 °C)	[67]
LaFe_0.475_Co_0.475_Pd_0.05_O_3_	0.072	1	5	7.2 (H_2_O) + 7.2 (CO_2_)	55,400	>50 (max. 85)	175–300	76 (at 250 °C)	[67]
La_0.8_Sr_0.2_Fe_0.9_Pd_0.1_O_3_	0.1	1	5	-	180,000	>50 (max. 96)	120–210	67 (at 160 °C)	[81]
BaTi_0.95_Pd_0.05_O_3_	0.072	1	5	7.2 (H_2_O) + 7.2 (CO_2_)	55,400	>50 (max. 92)	150–300	72 (at 200 °C)	[85]
BaTi_0.95_Pd_0.05_O_3_	0.045	0.8	5	-	1.61 × 10^6^	>50 (max. 55)	200–300	68 (at 250 °C)	[85]
Pd/BaTiO_3_	0.045	0.8	5	-	1.61 × 10^6^	>50 (max. 70)	125–250	60 (at 150 °C)	[85]
0.3%Pt/La_0.7_Sr_0.2_Ce_0.1_FeO_3_	0.25	1	5	-	40,000	>50 (max. 83)	125–225	93 (at 170 °C)	[9]
0.1%Pt/La_0.5_Ce_0.5_MnO_3_	0.25	1	5	5(H_2_O)	40,000	>50 (max. 88)	125–175	78 (at 150 °C)	[86]
Conventional, supported on oxide supports, NM catalysts									
1%Pt/SiO_2_	0.072	1	5	7.2 (H_2_O) + 7.2 (CO_2_)	55,400	>50 (max. 80)	100–175	51 (at 125 °C)	[67]
1%Pt/Al_2_O_3_	0.05	0.2	6	-	120,000	50	150	30	[82]
1%Pt/SiO_2_	0.05	0.2	6	-	120,000	>50 (max. 76)	85–110	20	[82]
0.5%Pt/Al_2_O_3_	0.05	0.4	5	-	120,000	>50 (max. 80)	100–225	60 (at 175 °C)	[87]
0.5%Pd/Al_2_O_3_	0.05	0.4	5	-	120,000	9	275	72	[87]
1%Pd/Al_2_O_3_	1	1	1		100,000	30	160	23	[88]
0.1%Pt/MgO-CeO_2_	0.25	1	5	5(H_2_O)	40,000	>50 (max. 95)	90–250	78 (at 150 °C)	[65]
0.5%Pd/Al_2_O_3_	0.1	0.75	6	0.25(CO)	240,000	30	210	70	[89]
0.5%Pd/Al_2_O_3_-(10%TiO_2_)	0.1	0.75	6	0.25(CO)	240,000	>50 (max. 92)	160–450	70 (at 265 °C)	[89]

**Table 3:** Representative literature for NO reduction by CO over perovskites and on transitional noble metal catalysts.

Catalyst	Reaction Conditions					Achievements			Ref.
	NO (%)	CO (%)	O_2_ (%)	Other (%)	WGHSV (mL/g∙h)	X_NO_ (%)	at T (°C)	max. S_N2_ (%)	
**Perovskite catalysts**									
La_0.6_Ce_0.4_FeO_3_ ^(a)^	0.04	0.05	-	-	24,000 h^−1^ (GHSV)	>50 (max. 88)	350–500	90 (at 350)	[90]
La_0.6_Ce_0.4_FeO_3_ ^(a)^	0.04	0.05	-	0.01 (SO_2_)	24,000 h^−1^ (GHSV)	76	500	n/a	[90]
La_0.6_Ce_0.4_FeO_3_ ^(a)^	0.04	0.05	3	0.01 (SO_2_) + 3 (H_2_O)	24,000 h^−1^ (GHSV)	>50 (max. 74)	350–500	n/a	[90]
LaCu_0.5_Mn_0.5_O_3_ ^(b)^	5	10	-	-	60,000	100	300–600	100	[91]
LaCu_0.25_Co_0.75_O_3_-750 ^(c)^	5	10	-	-	60,000	100	350–600	100	[92]
La_0.8_Ce_0.2_Cu_0.25_Co_0.75_O_3_ ^(d)^	5	10	-	-	60,000	100	290–600	100	[93]
LaNi_0.5_Cu_0.5_O_3_ ^(e)^	5	10	-	-	36,000	100	375–500	100 (at 450 °C)	[94]
LaMn_0.3_Fe_0.7_O_3_ ^(f)^	0.3	0.3	-	-	12,000 h^−1^ (GHSV)	90–100	390–450	90–100	[95]
La_0.8_Ce_0.2_Fe_0.7_Mn_0.3_O_3_ ^(g)^	0.3	0.3	-	-	12,000 h^−1^ (GHSV)	90–100	340–450	92–96	[95]
LaFe_0.5_Mn_0.5_O_3_ ^(h)^	0.3	0.3	-	-	12,000 h^−1^ (GHSV)	90–100	420–450	92–96	[96]
LaMn_0.5_Cu_0.5_O_3_^(i)^	0.3	0.3	-	-	12,000 h^−1^ (GHSV)	90–100	400–450	90–98	[96]
LaFeO_3_-nanocast	0.5	0.5	-	-	30,000	100	375–700	100	[99]
LaFeO_3_-uncast	0.5	0.5	-	-	30,000	100	600–700	100	[99]
LaFe_0.6_Co_0.4_O_3_-nanocast	0.5	0.5	-	-	30,000	100	550–700	100	[99]
LaFe_0.6_Co_0.4_O_3_-uncast	0.5	0.5	-	-	30,000	100	650–700	100	[99]
LaCo_0.5_Cu_0.5_O_3_ ^(j)^	4	4	-	-	150,000	95	400	n/a	[59]
LaCu_0.7_Mn_0.3_O_3_ ^(k)^	0.3	0.3	-	-	12,000 h^−1^ (GHSV)	>90	360–450	n/a	[35]
La_0.8_Sr_0.2_Cu_0.7_Mn_0.3_O_3_ ^(*l*)^	0.3	0.3	-	-	12,000 h^−1^ (GHSV)	>90	320–450	n/a	[35]
La_0.8_Ce_0.2_FeO_3_ ^(m)^	2	2	-	-	30,000	>90	330–500	n/a	[64]
LaMnO_3_ (r); LaFeO_3_ (r) *	2	2	-	-	30,000	>90	420–500	n/a	[100]
LaMnO_3_ ^(n)^	2	2	-	-	30,000	>90	510–570	100	[101]
**Conventional, supported on oxide supports, NM catalysts**									
0.5wt%Pt/γ-Al_2_O_3_	0.05	0.4	5	-	120,000	26	250	80	[87]
0.5wt%Pd/γ-Al_2_O_3_	0.05	0.4	5	-	120,000	13	180	62	[87]
0.5wt%Pt/γ-Al_2_O_3_	0.1	0.1	-	-	600,000	60	480	60	[102]
0.5wt%Pt(9.7%Rb)/γ-Al_2_O_3_	0.1	0.1	-	-	600,000	>90	320–500	100 (at 350 °C)	[102]
0.5wt%Rh/γ-Al_2_O_3_	0.1	0.1	-	-	600,000	>90	250–500	100 (at 300 °C)	[102]

^(a)^ The optimal from a series of La_x_Ce_1-x_FeO_3_ (x = 0.2, 0.4, 0.6, 0.8, 1) perovskites investigated. ^(b)^ The optimal from a series of LaM_0.5_Mn_0.5_O_3_ (M = Cu, Co, Fe, Ni, Cr) perovskites investigated. ^(c)^ The optimal from a LaCu_0.25_Co_0.75_O_3_ perovskite calcined at differed temperatures (250, 500, 750, and 1000 °C). ^(d)^ The optimal from a series of B-site partially substituted La_0.8_Ce_0.2_M_0.25_Co_0.75_O_3_ (M = Fe, Mn, Cu) perovskite. ^(e)^ The optimal from a series of LaNi_0.5_M_0.5_O_3_ (M = Co, Mn, Cu) perovskites investigated. ^(f)^ The optimal from a series of LaMn_1−x_Fe_x_O_3_ (x = 0, 0.3, 0.5, 0.7, 1) perovskites investigated. ^(g)^ The optimal from a series of La_0.8_M_0.2_Mn_0.3_Fe_0.7_O_3_ (M = Ce, Ba, Cs, Sr) perovskites investigated. ^(h)^ The optimal from a series of LaFe_0.5_M_0.5_O_3_ (M = Cu, Co, Mn, Fe) perovskites investigated. ^(i)^ The optimal from a series of LaMn_0.5_M_0.5_O_3_ (M = Cu, Co, Mn, Fe) perovskites investigated. ^(j)^ The optimal from a series of LaCo_1−x_Cu_x_O_3_ (x = 0, 0.1, 0.3, and 0.5) perovskites investigated. ^(k)^ The optimal from a series of LaCu_0.7_B_0.3_O_3_ (B = Mn, Fe, Co) perovskites investigated. ^(*l*)^ The optimal from a series of La_0.8_A_0.2_Cu_0.7_Mn_0.3_O_3_ (A = Rb, Sr, Cs, Ba) perovskites investigated. ^(m)^ The optimal from a series of La_x_M_1-x_FeO_3_ (M = Sr and/or Ce) perovskites investigated. * Materials prepared by a microemulsion method in the reverse (r) state. ^(n)^ The optimal from a series of La_x_Sr_1−x_MnO_3_ perovskites investigated.

## Data Availability

No new data were created or analyzed in this study. Data sharing is not applicable to this article.

## Abbreviations

BET	Brunauer, Emmett and Teller
CBM	Coal Bed Methane
DFT	Density Functional Theory
DLS	Dynamic Light Scattering
DRIFTS	Diffuse Reflectance Infrared Fourier Transform Spectroscopy
DSC	Differential Scanning Calorimetry
EDS	Energy-Dispersive X-ray Spectroscopy
EF-TEM	Energy Filtering Transmission Electron Microscopy
FE-SEM	Field Emission Scanning Electron Microscopy
FTIR	Fourier Transform Infrared Spectroscopy
HCs	Hydrocarbons
ICP-AES	Inductively Coupled Plasma Atomic Emission Spectroscopy
ICP-OES	Inductively Coupled Plasma Optical Emission Spectroscopy
PM	Particulate Matter
SCR	Selective Catalytic Reduction
SEM	Scanning Electron Microscopy
TEM	Transmission Electron Microscopy
TPD	Temperature-Programmed Desorption
TPR	Temperature-Programmed Reduction
TWC	Three-Way Catalysts
WGHSV	Weight-basis Gas Hourly Space Velocity
XRD	X-ray Diffraction
XPS	X-ray Photoelectron Spectroscopy

## References

[1] Granger P., Parvulescu V.I. (2011). Catalytic NOx abatement systems for mobile sources: From three-way to lean burn after-treatment technologies. Chem. Rev..

[2] Yentekakis I.V., Konsolakis M. (2016). Three-way Catalysis. Perovskites and Related Mixed Oxides.

[3] Yentekakis I.V., Vernoux P. (2019). Emissions control catalysis. Catalysts.

[4] Yentekakis I.V., Vernoux P., Goula G., Caravaca A. (2019). Electropositive promotion by Alkalis or Alkaline earths of Pt-group metals in emissions control catalysis: A status report. Catalysts.

[5] Yentekakis I.V., Dong F. (2020). Grand Challenges for Catalytic Remediation in Environmental and Energy Applications Toward a Cleaner and Sustainable Future. Front. Environ. Chem..

[6] Damma D., Ettireddy P.R., Reddy B.M., Smirniotis P.G. (2019). A review of low temperature NH_3_-SCR for removal of NOx. Catalysts.

[7] Yentekakis I.V., Tellou V., Botzolaki G., Rapakousios I.A. (2005). A comparative study of the C_3_H_6_ + NO + O_2_, C_3_H_6_ + O_2_ and NO + O_2_ reactions in excess oxygen over Na-modified Pt/γ-Al_2_O_3_ catalysts. Appl. Catal. B Environ..

[8] Goula M.A., Charisiou N.D., Papageridis K.N., Delimitis A., Papista E., Pachatouridou E., Iliopoulou E.F., Marnellos G., Konsolakis M., Yentekakis I.V. (2016). A comparative study of the H_2_-assisted selective catalytic reduction of nitric oxide by propene over noble metal (Pt, Pd, Ir)/γ-Al_2_O_3_ catalysts. J. Environ. Chem. Eng..

[9] Costa C.N., Savva P.G., Andronikou C., Lambrou P.S., Polychronopoulou K., Belessi V.C., Stathopoulos V.N., Pomonis P.J., Efstathiou A.M. (2002). An investigation of the NO/H_2_/O_2_ (Lean De-NOx) reaction on a highly active and selective Pt/La_0.7_Sr_0.2_Ce_0.1_FeO_3_ catalyst at low temperatures. J. Catal..

[10] Polychronopoulou K., Efstathiou A.M. (2012). NOx Control via H_2_-Selective Catalytic Reduction (H_2_-SCR) Technology for Stationary and Mobile Applications. Recent Patents Mater. Sci..

[11] Machida M., Ikeda S., Kurogi D., Kijima T. (2001). Low temperature catalytic NOx–H_2_ reactions over Pt/TiO_2_-ZrO_2_ in an excess oxygen. Appl. Catal. B Environ..

[12] Macleod N., Lambert R.M. (2003). An in situ DRIFTS study of efficient lean NOx reduction with H_2_ + CO over Pd/Al_2_O_3_: The key role of transient NCO formation in the subsequent generation of ammonia. Appl. Catal. B Environ..

[13] Konsolakis M., Vrontaki M., Avgouropoulos G., Ioannides T., Yentekakis I.V. (2006). Novel doubly-promoted catalysts for the lean NOx reduction by H_2_ + CO: Pd(K)/Al_2_O_3_–(TiO_2_). Appl. Catal. B Environ..

[14] Pekridis G., Kaklidis N., Komvokis V., Athanasiou C., Konsolakis M., Yentekakis I.V., Marnellos G.E. (2010). Surface and catalytic elucidation of Rh/γ-Al_2_O_3_ catalysts during NO reduction by C_3_H_8_ in the presence of excess O_2_, H_2_O, and SO_2_. J. Phys. Chem. A.

[15] Burch R. (2004). Knowledge and know-how in emission control for mobile applications. Catal. Rev. Sci. Eng..

[16] Macleod N., Isaac J., Lambert R.M. (2000). Sodium Promotion of the NO+C_3_H_6_ Reaction over Rh/γ-Al_2_O_3_ Catalysts. J. Catal..

[17] Yentekakis I.V., Konsolakis M., Rapakousios I.A., Matsouka V. (2007). Novel electropositively promoted monometallic (Pt-only) catalytic converters for automotive pollution control. Top. Catal..

[18] Yentekakis I.V., Lambert R.M., Konsolakis M., Kiousis V. (1998). The effect of sodium on the Pd-catalyzed reduction of NO by methane. Appl. Catal. B Environ..

[19] Konsolakis M., Yentekakis I.V. (2001). Strong promotional effects of Li, K, Rb and Cs on the Pt-catalysed reduction of NO by propene. Appl. Catal. B Environ..

[20] Konsolakis M., Yentekakis I.V. (2001). The reduction of NO by propene over Ba-promoted Pt/γ-Al_2_O_3_ catalysts. J. Catal..

[21] Tanikawa K., Egawa C. (2011). Effect of barium addition over palladium catalyst for CO-NO-O_2_ reaction. J. Mol. Catal. A Chem..

[22] Palermo A., Lambert R.M., Harkness I.R., Yentekakis I.V., Marina O., Vayenas C.G. (1996). Electrochemical promotion by Na of the platinum-catalyzed reaction between CO and NO. J. Catal..

[23] Papadakis V.G., Pliangos C.A., Yentekakis I.V., Verykios X.E., Vayenas C.G. (1996). Development of high performance, Pd-based, three-way catalysts. Catal. Today.

[24] Palermo A., Tikhov M.S., Filkin N.C., Lambert R.M., Yentekakis I.V., Vayenas C.G. (1996). Electrochemical promotion of NO reduction by CO and by propene. Stud. Surf. Sci. Catal..

[25] Matsouka V., Konsolakis M., Yentekakis I.V., Papavasiliou A., Tsetsekou A., Boukos N. (2011). Thermal aging behavior of Pt-only TWC converters under simulated exhaust conditions: Effect of rare earths (CeO_2_, La_2_O_3_) and alkali (Na) modifiers. Top. Catal..

[26] Yentekakis I.V., Goula G., Panagiotopoulou P., Kampouri S., Taylor M.J., Kyriakou G., Lambert R.M. (2016). Stabilization of catalyst particles against sintering on oxide supports with high oxygen ion lability exemplified by Ir-catalyzed decomposition of N_2_O. Appl. Catal. B Environ..

[27] Yentekakis I.V., Goula G., Kampouri S., Betsi-Argyropoulou I., Panagiotopoulou P., Taylor M.J., Kyriakou G., Lambert R.M. (2018). Ir-Catalysed Nitrous oxide (N_2_O) Decomposition: Effect of Ir Particle Size and Metal–Support Inter-actions. Catal. Letters.

[28] Yentekakis I.V., Goula G., Panagiotopoulou P., Katsoni A., Diamadopoulos E., Mantzavinos D., Delimitis A. (2015). Dry reforming of methane: Catalytic performance and stability of Ir catalysts supported on γ-Al_2_O_3_, Zr_0.92_Y_0.08_O_2-δ_ (YSZ) or Ce_0.9_Gd_0.1_O_2-δ_ (GDC) supports. Top. Catal..

[29] Goula G., Botzolaki G., Osatiashtiani A., Parlett C.M.A., Kyriakou G., Lambert R.M., Yentekakis I.V. (2019). Oxidative thermal sintering and redispersion of Rh nanoparticles on supports with high oxygen ion lability. Catalysts.

[30] Nikolaraki E., Goula G., Panagiotopoulou P., Taylor M.J., Kousi K., Kyriakou G., Kondarides D.I., Lambert R.M., Yentekakis I.V. (2021). Support induced effects on the Ir nanoparticles activity, selectivity and stability performance under CO_2_ reforming of methane. Nanomaterials.

[31] Parvulescu V.I., Kaliaguine S., Prellier W. (2016). Perovskites and Related Mixed Oxides.

[32] Zhu J., Li H., Zhong L., Xiao P., Xu X., Yang X., Zhao Z., Li J. (2014). Perovskite oxides: Preparation, characterizations, and applications in heterogeneous catalysis. ACS Catal..

[33] Royer S., Duprez D., Can F., Courtois X., Batiot-Dupeyrat C., Laassiri S., Alamdari H. (2014). Perovskites as substitutes of noble metals for heterogeneous catalysis: Dream or reality. Chem. Rev..

[34] Hwang J., Rao R.R., Giordano L., Katayama Y., Yu Y., Shao-Horn Y. (2017). Perovskites in catalysis and electrocatalysis. Science.

[35] Tarjomannejad A., Niaei A., Gómez M.J.I., Farzi A., Salari D., Albaladejo-Fuentes V. (2017). NO + CO reaction over LaCu_0.7_B_0.3_O_3_ (B = Mn, Fe, Co) and La_0.8_A_0.2_Cu_0.7_Mn_0.3_O_3_ (A = Rb, Sr, Cs, Ba) perovskite-type catalysts. J. Therm. Anal. Calorim..

[36] Bhattar S., Abedin M.A., Kanitkar S., Spivey J.J. (2021). A review on dry reforming of methane over perovskite derived catalysts. Catal. Today.

[37] Sim Y., Kwon D., An S., Ha J.M., Oh T.S., Jung J.C. (2020). Catalytic behavior of ABO_3_ perovskites in the oxidative coupling of methane. Mol. Catal..

[38] Yang E.-h., Noh Y.S., Hong G.H., Moon D.J. (2018). Combined steam and CO_2_ reforming of methane over La_1-x_Sr_x_NiO_3_ perovskite oxides. Catal. Today.

[39] Lima S.M., Assaf J.M., Peña M.A., Fierro J.L.G. (2006). Structural features of La_1-x_Ce_x_NiO_3_ mixed oxides and performance for the dry reforming of methane. Appl. Catal. A Gen..

[40] Wang M., Zhao T., Dong X., Li M., Wang H. (2018). Effects of Ce substitution at the A-site of LaNi_0.5_Fe_0.5_O_3_ perovskite on the enhanced catalytic activity for dry reforming of methane. Appl. Catal. B Environ..

[41] Peña M.A., Fierro J.L.G. (2001). Chemical structures and performance of perovskite oxides. Chem. Rev..

[42] Shen M., Zhao Z., Chen J., Su Y., Wang J., Wang X. (2013). Effects of calcium substitute in LaMnO_3_ perovskites for NO catalytic oxidation. J. Rare Earths.

[43] Zhang R., Villanueva A., Alamdari H., Kaliaguine S. (2006). Reduction of NO by CO over nanoscale LaCo_1-x_Cu_x_O_3_ and LaMn_1-x_Cu_x_O_3_ perovskites. J. Mol. Catal. A Chem..

[44] Misono M. (2005). A view on the future of mixed oxide catalysts: The case of heteropolyacids (polyoxometalates) and perovskites. Catal. Today.

[45] Yentekakis I.V. (2006). Open- and closed-circuit study of an intermediate temperature SOFC directly fueled with simulated biogas mixtures. J. Power Sources.

[46] Yentekakis I.V., Papadam T., Goula G. (2008). Electricity production from wastewater treatment via a novel biogas-SOFC aided process. Solid State Ionics.

[47] Buciuman F.-C., Joubert E., Menezo J.-C., Barbier J. (2001). Catalytic properties of La_0.8_A_0.2_MnO_3_ (A = Sr, Ba, K, Cs) and LaMn_0.8_B_0.2_O_3_ (B = Ni, Zn, Cu) perovskites. Appl. Catal. B Environ..

[48] Wu X., Xu L., Weng D. (2004). The NO selective reduction on the La_1-x_Sr_x_MnO_3_ catalysts. Catal. Today.

[49] Kousi K., Tang C., Metcalfe I.S., Neagu D. (2021). Emergence and future of exsolved materials. Small.

[50] Nishihata Y., Mizuki J., Akao T., Tanaka H., Uenishi M., Kimura M., Okamoto T., Hamada N. (2002). Self-regeneration of a Pd-perovskite catalyst for automotive emissions control. Nature.

[51] Kwon O., Joo S., Choi S., Sengodan S., Kim G. (2020). Review on exsolution and its driving forces in perovskites. J. Phys Energy.

[52] He H., Dai H.X., Au C.T. (2001). An investigation on the utilization of perovskite-type oxides La_1-x_Sr_x_MO_3_ (M = Co_0.77_Bi_0.20_Pd_0.03_) as three-way catalysts. Appl. Catal. B Environ..

[53] Zhu J., Zhao Z., Xiao D., Li J., Yang X., Wu Y. (2005). Study of La_2-x_Sr_x_CuO_4_ (x = 0.0, 0.5, 1.0) catalysts for NO + CO reaction from the measurements of O_2_-TPD, H_2_-TPR and cyclic voltammetry. J. Mol. Catal. A Chem..

[54] Fino D., Fino P., Saracco G., Specchia V. (2003). Studies on kinetics and reactions mechanism of La_2-x_K_x_Cu_1-y_VyO_4_ layered perovskites for the combined removal of diesel particulate and NOx. Appl. Catal. B Environ..

[55] Centi G., Perathoner S. (1995). Nature of active species in copper-based catalysts and their chemistry of transformation of nitrogen oxides. Appl. Catal. A Gen..

[56] Yahiro H., Iwamoto M. (2001). Copper ion-exchanged zeolite catalysts in deNOx reaction. Appl. Catal. A Gen..

[57] Zhang R., Villanueva A., Alamdari H., Kaliaguine S. (2006). Catalytic reduction of NO by propene over LaCo_1-x_Cu_x_O_3_ perovskites synthesized by reactive grinding. Appl. Catal. B Environ..

[58] Zhang R., Villanueva A., Alamdari H., Kaliaguine S. (2006). SCR of NO by propene over nanoscale LaMn_1-x_Cu_x_O_3_ perovskites. Appl. Catal. A Gen..

[59] Glisenti A., Pacella M., Guiotto M., Natile M.M., Canu P. (2016). Largely Cu-doped LaCo_1-x_Cu_x_O_3_ perovskites for TWC: Toward new PGM-free catalysts. Appl. Catal. B Environ..

[60] Levasseur B., Kaliaguine S. (2009). Effects of iron and cerium in La_1-y_Ce_y_Co_1-x_Fe_x_O_3_ perovskites as catalysts for VOC oxidation. Appl. Catal. B Environ..

[61] Deng C., Huang Q., Zhu X., Hu Q., Su W., Qian J., Dong L., Li B., Fan M., Liang C. (2016). The influence of Mn-doped CeO_2_ on the activity of CuO/CeO_2_ in CO oxidation and NO + CO model reaction. Appl. Surf. Sci..

[62] Deng C., Qian J., Yu C., Yi Y., Zhang P., Li W., Dong L., Li B., Fan M. (2016). Influences of doping and thermal stability on the catalytic performance of CuO/Ce_20_M_1Ox_ (M = Zr, Cr, Mn, Fe, Co, Sn) catalysts for NO reduction by CO. RSC Adv..

[63] Ma J., Jin G., Gao J., Li Y., Dong L., Huang M., Huang Q., Li B. (2015). Catalytic effect of two-phase intergrowth and coexistence CuO-CeO_2_. J. Mater. Chem. A.

[64] Giannakas A.E., Leontiou A.A., Ladavos A.K., Pomonis P.J. (2006). Characterization and catalytic investigation of NO + CO reaction on perovskites of the general formula La_x_M_1-x_FeO_3_ (M = Sr and/or Ce) prepared via a reverse micelles microemulsion route. Appl. Catal. A Gen..

[65] Costa C.N., Efstathiou A.M. (2007). Low-temperature H_2_-SCR of NO on a novel Pt/MgO-CeO_2_ catalyst. Appl. Catal. B Environ..

[66] Engelmann-Pirez M., Granger P., Leclercq G. (2005). Investigation of the catalytic performances of supported noble metal based catalysts in the NO + H_2_ reaction under lean conditions. Catal. Today.

[67] Mondragón Rodríguez G.C., Saruhan B. (2010). Effect of Fe/Co-ratio on the phase composition of Pd-integrated perovskites and its H_2_-SCR of NOx performance. Appl. Catal. B Environ..

[68] Sato S., Yu-u Y., Yahiro H., Mizuno N., Iwamoto M. (1991). Cu-ZSM-5 zeolite as highly active catalyst for removal of nitrogen monoxide from emission of diesel engines. Appl. Catal..

[69] Vasala S., Karppinen M. (2015). A_2_*B′B″*O_6_ perovskites: A review. Prog. Solid State Chem..

[70] Li X., Chen C., Liu C., Xian H., Guo L., Lv J., Jiang Z. (2013). Pd-Doped Perovskite: An Effective Catalyst for Removal of NO. ACS Catal..

[71] Kucherov A.V., Gerlock J.L., Jen H.W., Shelef M. (1995). In Situ ESR Monitoring of CuH-ZSM-5 Up to 500 °C in Flowing Dry Mixtures of NO(NO_2_), C_3_H_6_(C_2_H_5_OH), and Excess O_2_. J. Catal..

[72] Ukisu Y., Miyadera T., Abe A., Yoshida K. (1996). Infrared study of catalytic reduction of lean NOx with alcohols over alumina-supported silver catalyst. Catal. Letters.

[73] Wu Q., He H., Yu Y. (2005). In situ DRIFTS study of the selective reduction of NOx with alcohols over Ag/Al_2_O_3_ catalyst: Role of surface enolic species. Appl. Catal. B Environ..

[74] Wang H., Zhang R., Li P., Royer S., Dacquin J.P. (2019). Mechanistic insight into the methanol selective catalytic reduction of NO reaction over Cu-containing perovskites. J. Catal..

[75] Teng Z., Huang S., Zhang H., Yu H., Li N., Zhou Q. (2018). A system including enriching coal bed methane by solar energy and selective catalytic reduction. Appl. Therm. Eng..

[76] Teng Z., Zhang H., Huang S., Li N., Zhou Q. (2018). Experimental study on reduction of NO by CH_4_ over La_0.8_Sr_0.2_MnO_3_/α-Al_2_O_3_ in excess of O_2_. J. Taiwan Inst. Chem. Eng..

[77] Giroir-Fendler A., Gil S., Baylet A. (2014). (La_0.8_A_0.2_)MnO_3_ (A = Sr, K) perovskite catalysts for NO and C_10_H_22_ oxidation and selective reduction of NO by C_10_H_22_. Cuihua Xuebao Chin. J. Catal..

[78] Tabata K., Hirano Y., Suzuki E. (1998). XPS studies on the oxygen species of LaMn_1-x_Cu_x_O_3+λ_. Appl. Catal. A Gen..

[79] Baylet A., Royer S., Labrugère C., Valencia H., Marécot P., Tatibouët J.M., Duprez D. (2008). Effect of palladium on the reducibility of Mn based materials: Correlation with methane oxidation activity. Phys. Chem. Chem. Phys..

[80] Luo Y., Wang X., Qian Q., Chen Q. (2014). Studies on B sites in Fe-doped LaNiO_3_ perovskite for SCR of NOx with H_2_. Int. J. Hydrogen Energy.

[81] Furfori S., Russo N., Fino D., Saracco G., Specchia V. (2010). NO SCR reduction by hydrogen generated in line on perovskite-type catalysts for automotive diesel exhaust gas treatment. Chem. Eng. Sci..

[82] Burch R., Coleman M.D. (1999). An investigation of the NO/H_2_/O_2_ reaction on noble-metal catalysts at low temperatures under lean-burn conditions. Appl. Catal. B Environ..

[83] Dhainaut F., Pietrzyk S., Granger P. (2007). Kinetic investigation of the NO reduction by H_2_ over noble metal based catalysts. Catal. Today.

[84] Barrera A., Viniegra M., Bosch P., Lara V.H., Fuentes S. (2001). Pd/Al_2_O_3_-La_2_O_3_ catalysts prepared by sol-gel: Characterization and catalytic activity in the NO reduction by H_2_. Appl. Catal. B Environ..

[85] Mondragon Rodriguez G.C., Kelm K., Saruhan B. (2010). H_2_-selective catalytic reduction of NOx activity and microstructural analysis of new BaTi_0.9_5Pd_0.0_5O_3_ catalyst. Appl. Catal. A Gen..

[86] Costa C.N., Efstathiou A.M., Stathopoulos V.N., Belessi V.C. (2001). An investigation of the NO/H_2_/O_2_ (Lean-deNOx) reaction on a highly active and selective Pt/La_0.5_Ce_0.5_MnO_3_ catalyst. J. Catal..

[87] Macleod N., Lambert R.M. (2002). Lean NOx reduction with CO + H_2_ mixtures over Pt/Al_2_O_3_ and Pd/Al_2_O_3_ catalysts. Appl. Catal. B Environ..

[88] Dhainaut F., Pietrzyk S., Granger P. (2007). NO + H_2_ reaction on Pd/Al_2_O_3_ under lean conditions: Kinetic study. Top. Catal..

[89] Matsouka V., Konsolakis M., Lambert R.M., Yentekakis I.V. (2008). In situ DRIFTS study of the effect of structure (CeO_2_–La_2_O_3_) and surface (Na) modifiers on the catalytic and surface behaviour of Pt/γ-Al_2_O_3_ catalyst under simulated exhaust conditions. Appl. Catal. B Environ..

[90] Qin Y., Sun L., Zhang D., Huang L. (2016). Role of ceria in the improvement of SO_2_ resistance of La_x_Ce_1-X_FeO_3_ catalysts for catalytic reduction of NO with CO. Catal. Commun..

[91] Wu Y., Liu H., Li G., Jin L., Li X., Ou X., Dong L., Jin G., Li B. (2020). Tuning composition on B sites of LaM_0.5_Mn_0.5_O_3_ (M = Cu, Co, Fe, Ni, Cr) perovskite catalysts in NOx efficient reduction. Appl. Surf. Sci..

[92] Wu Y., Chu B., Zhang M., Yi Y., Dong L., Fan M., Jin G., Zhang L., Li B. (2019). Influence of calcination temperature on the catalytic properties of LaCu_0.25_Co_0.75_O_3_ catalysts in NOx reduction. Appl. Surf. Sci..

[93] Wu Y., Li G., Chu B., Dong L., Tong Z., He H., Zhang L., Fan M., Li B., Dong L. (2018). NO Reduction by CO over Highly Active and Stable Perovskite Oxide Catalysts La_0.8_Ce_0.2_M_0.25_Co_0.75_O_3_ (M = Cu, Mn, Fe): Effect of the Role in B Site. Ind. Eng. Chem. Res..

[94] Yi Y., Liu H., Chu B., Qin Z., Dong L., He H., Tang C., Fan M., Bin L. (2019). Catalytic removal NO by CO over La-Ni_0.5_M_0.5_O_3_ (M = Co, Mn, Cu) perovskite oxide catalysts: Tune surface chemical composition to improve N_2_ selectivity. Chem. Eng. J..

[95] Tarjomannejad A., Farzi A., Gómez M.J.I., Niaei A., Salari D., Albaladejo-Fuentes V. (2016). Catalytic Reduction of NO by CO over LaMn_1−x_Fe_x_O_3_ and La_0.8_A_0.2_Mn_0.3_Fe_0.7_O_3_ (A = Sr, Cs, Ba, Ce) Perovskite Catalysts. Catal. Letters.

[96] Tarjomannejad A., Farzi A., Niaei A., Salari D. (2017). NO reduction by CO over LaB_0.5_B′_0.5_O_3_ (B = Fe, Mn, B′ = Fe, Mn, Co, Cu) perovskite catalysts, an experimental and kinetic study. J. Taiwan Inst. Chem. Eng..

[97] Lorimer D., Bell A.T. (1979). Reduction of NO by CO over a silica-supported platinum catalyst: Infrared and kinetic studies. J. Catal..

[98] Zhdanov V.P., Kasemo B. (1997). Mechanism and kinetics of the NO-CO reaction on Rh. Surf. Sci. Rep..

[99] De Lima R.K.C., Batista M.S., Wallau M., Sanches E.A., Mascarenhas Y.P., Urquieta-González E.A. (2009). High specific surface area LaFeCo perovskites-Synthesis by nanocasting and catalytic behavior in the reduction of NO with CO. Appl. Catal. B Environ..

[100] Giannakas A.E., Ladavos A.K., Pomonis P.J. (2004). Preparation, characterization and investigation of catalytic activity for NO + CO reaction of LaMnO_3_ and LaFeO_3_ perovskites prepared via microemulsion method. Appl. Catal. B Environ..

[101] Leontiou A.A., Ladavos A.K., Armatas G.S., Trikalitis P.N., Pomonis P.J. (2004). Kinetics investigation of NO + CO reaction on La-Sr-Mn-O perovskite-type mixed oxides. Appl. Catal. A Gen..

[102] Konsolakis M., Yentekakis I.V., Palermo A., Lambert R.M. (2001). Optimal promotion by rubidium of the CO + NO reaction over Pt/γ-Al_2_O_3_ catalysts. Appl. Catal. B Environ..

